# Impact of Citrus Pulp or Inulin on Intestinal Microbiota and Metabolites, Barrier, and Immune Function of Weaned Piglets

**DOI:** 10.3389/fnut.2021.650211

**Published:** 2021-12-03

**Authors:** Julie Uerlings, Ester Arévalo Sureda, Martine Schroyen, Kikianne Kroeske, Sofie Tanghe, Maartje De Vos, Geert Bruggeman, José Wavreille, Jérôme Bindelle, Giorgia Purcaro, Nadia Everaert

**Affiliations:** ^1^Precision Livestock and Nutrition Unit, TERRA Teaching and Research Center, Gembloux Agro-Bio Tech, University of Liège, Gembloux, Belgium; ^2^Research Foundation for Industry and Agriculture, National Scientific Research Foundation (FRIA-FNRS), Brussels, Belgium; ^3^Flanders Research Institute for Agriculture, Fisheries and Food (ILVO), Melle, Belgium; ^4^Royal Agrifirm Group, Apeldoorn, Netherlands; ^5^Production and Sectors Department, Walloon Agricultural Research Center, Gembloux, Belgium; ^6^Analytical Chemistry Lab, TERRA Teaching and Research Center, Gembloux Agro-Bio Tech, University of Liège, Gembloux, Belgium; ^7^Animal and Human Health Engineering, Department of Biosystems, Katholieke Universiteit Leuven, Heverlee, Belgium

**Keywords:** citrus pulp, inulin, intestinal health, inflammation, barrier function, gut morphology, microbiota, metabolites

## Abstract

We investigated the use of citrus pulp (CP) as a novel prebiotic capable of exerting microbiota and immunomodulating capacities to alleviate weaning stress. Inulin (IN), a well-known prebiotic, was used for comparison. Hundred and 28 male weaned piglets of 21 days old were assigned to 32 pens of 4 piglets each. Piglets were assigned to one of the four treatments, i.e., control, IN supplemented at 0.2% (IN0.2%), and CP supplemented either at 0.2% (CP0.2%) or at 2% (CP2%). On d10–11 and d31–32 post-weaning, one pig per pen was euthanized for intestinal sampling to evaluate the growth performance, chyme characteristics, small intestinal morphology, colonic inflammatory response and barrier integrity, metabolite profiles [gas chromatography-mass spectrometry (GC-MS), and liquid chromatography-mass spectrometry (LC-MS)], and microbial populations. The IN treatment and the two CP treatments induced higher small intestinal villus height to crypt depth ratios in comparison with the control diet at both sampling times. All treatments decreased acidic goblet cell absolute counts in the crypts in comparison to the control diet of the duodenum on d10–11 and d31–32. The gene expression of β-defensin 2 was downregulated in colonic tissues following the IN and CP2% inclusion on d31–32. On d31–32, piglets fed with IN and CP0.2% showed lower mRNA levels of occludin and claudin-3, respectively. Not surprisingly, flavonoids were observed in the colon in the CP treatments. Increased colonic acetate proportions on d10–11, at the expense of branched-chain fatty acid (BCFA) levels, were observed following the CP2% supplementation compared to the control diet, inferring a reduction of proteolytic fermentation in the hindgut. The beneficial microbial community *Faecalibacterium* spp. was promoted in the colon of piglets fed with CP2% on d10–11 (*p* = 0.04; false discovery rate (FDR) non-significant) and on d31–32 (*p* = 0.03; FDR non-significant) in comparison with the control diet. Additionally, on d31–32, CP2% increased the relative abundance of *Megasphaera* spp. compared to control values (*p* = 0.03; FDR non-significant). In conclusion, CP2% promoted the growth of beneficial bacterial communities in both post-weaning time points, modulating colonic fermentation patterns in the colon. The effects of CP supplementation were similar to those of IN and showed the potential as a beneficial feed supplement to alleviate weaning stress.

## Introduction

In the pig industry, the selection for hyper-prolific sows has caused higher mortality and morbidity in early life due to low body weight at birth, and more competition for suckling milk (1). For mammals, lactation is essential for appropriate postnatal gut development, especially in pigs due to the lack of maternal transfer of immunity *via* the placenta. In pig production, there is a tendency for shortening the lactation period with the objective to increase sow productivity by reducing the estrus interval, which results in an early and abrupt transition for piglets from a milk-based diet to a solid-feed diet. Indeed, weaning is a critical event in swine production comprising physiological, social, nutritional, and environmental challenges that result in detrimental changes in gut morphology, physiology, immunology, and function (2, 3). Moreover, as the mucosal immune system is still immature and a stable commensal microbiota is not yet established (4), this period is often associated with disturbed microbiota and the proliferation of pathogens in the hindgut, resulting in post-weaning diarrhea (5, 6). In addition, it has been demonstrated that weaning is associated with pro-inflammatory events in the host hindgut (7) and the marked changes in intestinal histology and barrier integrity (8).

Weaning stress can have an impact on the intestinal wall architecture leading to villus atrophy and crypt hyperplasia, which further compromises the digestive, absorptive, and secretory capacities of the small intestine (9). Besides, weaning coincides with a disruption of the intestinal barrier function with a loss of integrity (10, 11); as well as a destabilization of the local immune system and mucosal inflammation (12, 13). The composition and diversity of the gut microbiota as well as its metabolic activities are highly influenced by an abrupt change of diet. For instance, a rapid decline in *Lactobacillus* spp. is observed during the weaning transition, while *Clostridium* spp., *Prevotella* spp., *Proteobacteriaceae*, and *E*. c*oli* are increased (14). The shift of microbiota carries compositional and functional instabilities that often lead to dysbiosis, and consequently, to post-weaning disorders.

To overcome post-weaning losses and limit the use of antimicrobial compounds, dietary inclusion of functional ingredients and supplements remains an interesting strategy in pig production although their modes of action on gut health are still unclear (15–17) and chiefly depend on the specific composition. The functional activities of prebiotic ingredients include the stimulation of microbial fermentation, the proliferation of commensal bacterial communities, and the production of metabolites, such as short-chain fatty acids (SCFAs), attenuating hindgut protein fermentation (18), and conferring health-related benefits to the host (19, 20).

Citrus pulp (CP), including peels, internal tissues, and seeds, contains large amounts of soluble carbohydrates, including pectin (21, 22), which is extensively fermented by the hindgut microbiota (23, 24). Although CP has been primarily used in ruminant nutrition (21), it may also be considered as an alternative prebiotic in the weaned pigs (25–27). We hypothesized that the dried CP might have prebiotic activities similar to those of inulin (IN), a soluble fiber-rich fraction from the chicory sector (28), and a well-known feed supplement used in swine nutrition (29). Dietary IN has been shown to exert beneficial effects in the weaned piglets, including the stimulation of the immune system and the modulation of beneficial bacterial communities in the hindgut (29, 30). Therefore, the aim of the current study was to investigate the dose-dependent prebiotic potential of CP (CP0.2% and CP2%) next to IN (IN0.2%) in comparison with a basal control feed, devoid of health-modulating supplements, in terms of microbial, metabolomic, and immunomodulatory capacities during the post-weaning period.

## Materials and Methods

### Experiment 1

#### Animals and Diets

All experimental procedures on piglets were in accordance with European and Belgian regulations concerning the care and use of animals for research purposes and were performed at the facilities of the Nuscience Group (Melle, Belgium) under the license number A04/357 granted by the ethical commission of the University of Ghent to perform animal experiments.

A total of 128 male Large White × Danish Landrace × Piétrain piglets weaned at 21 days and with an average weight of 5.2 ± 0.5 kg (mean ± SD) were obtained from a commercial farm (Flanders, Belgium). Only male piglets were considered in this study to limit sex disparity in immunological function and susceptibility (31). The animals were allocated to 32 pens of 4 littermates based on body weight and were fed *ad libitum* during a 5-day adaptation period with a commercially formulated creep feed diet (Babistar Flex©, Nuscience Group, Melle, Belgium). Piglets were housed in pens with fully slatted plastic floors and wire-mesh sides in a temperature-controlled animal house, including eight identical rooms (with four pens each). Each pen was equipped with a feeder and a drinking bowl. Following the 5-day adaptation period, piglets were assigned to one of the four treatments (*n* = 8 pens): a control diet, an IN diet (control feed additionally supplemented with 0.2% of IN; Cosucra Group Warcoing SA, Warcoing, Belgium), and two CP diets (control feed additionally supplemented with either 0.2% or 2% of the dried CP (CP0.2/CP2%); Nuscience Group, Melle, Belgium). The dose of IN was chosen as suggested by the producer and based on the previous studies found in the literature that used 0.4–0.6% IN supplementation (32, 33). Moreover, Metzler-Zebeli et al. (34) showed that 0.2% IN was the median dose used in overall 43 studies, which was used in this study as a minimal effective dose. Therefore, the same dose was chosen for CP, and 10 times higher dose to reach similar amounts as described in few earlier studies (25–27). In each of the eight rooms, there was one pen per experimental diet. The basal diet was formulated to reach the nutritional requirements of animals following the NRC recommendations (Table 1). For the chemical composition of IN and CP, we refer to Uerlings et al. (35). Throughout the experiment, the pigs had *ad libitum* access to feed and water.

**Table 1:** Ingredient proportions of the control diet^*^ and analyzed chemical composition.

**INGREDIENTS** (%)	
**Barley**	**28.0**
**Corn heat treated**	**16.4**
**Wheat**	**15.0**
**Wheat heat treated**	**10.0**
**Soybean Danex**	**13.1**
**Soybean protein concentrate**	**4.0**
**Soybean meal**	**3.0**
**Soybean oil**	**1.5**
**DL-Methionine**	**0.24**
**L-Lysine**	**0.62**
**L-Threonine**	**0.29**
**L-Tryptophan**	**0.09**
**L-Valine**	**0.13**
**Salt**	**0.50**
**Monocalcium phosphate**	**0.73**
**Limestone**	**0.40**
Premix^**^	**6.0**
**NUTRIENT COMPOSITION**	
**Dry matter (%)**	**89.8**
**Crude protein (%)**	**16.9**
**Crude fat (%)**	**5.8**
**Crude ash (%)**	**4.6**
**Crude fiber (%)**	**4.7**
**Starch (%)**	**41.5**
**Sugars (%)**	**5.8**
**Calcium (%)**	**0.4**
**Phosphorus (%)**	**0.4**
**Sodium (%)**	**0.2**
**Zinc (%)**	**0.8**
**Manganese (%)**	**0.8**
**Iron (%)**	**2.8**
**Cupper (%)**	**1.0**

* *IN, CP0.2%, and CP2% treatments were formulated accordingly as the small levels of inclusion of the tested ingredients did not modify the analyzed chemical composition of the diets*.

** *The premix contained vitamins, trace elements, flavoring compounds, enzymes, and MCFA, providing the following quantities per kg of diet: vitamin A, 15,000 IU; vitamin D3, 2,000 IU; vitamin E, 110 mg; vitamin K3, 3 mg; vitamin B1, 1.5 mg; vitamin B2, 5.4 mg; calcium D-pantothenate, 17.9 mg; vitamin B6, 2.9 mg; vitamin B12, 0.04 mg; nicotinamide, 30.3 mg; choline chloride, 750 mg; iron(II) sulfate monohydrate, 120 mg; copper(II) sulfate pentahydrate, 150 mg; zinc sulfate monohydrate, 75 mg; zinc chelate of glycine hydrate, 25 mg; manganese(II) oxide, 80 mg; calcium iodate, 1 mg; sodium selenite, 0.35 mg; endo-1,4-beta-glucanase, 250 TGU; endo-1,4-beta-xylanase, 560 TXU; 6-phytase, 1,000 FYT*.

Six piglets facing too severe weight loss or with leg injuries after weaning had to be treated with antibiotics while one piglet suddenly died. Therefore, these were excluded from the experiment without affecting the number of experimental units, reaching eight pens per treatment for zootechnical performance parameters and eight piglets per treatment for the other parameters. A power analysis was performed using G^*^Power software to calculate the sample size required for the experiment.

#### Feed Chemical Analyses

Moisture, crude ash, crude fiber, crude protein, crude fat, sugar, and starch contents in the feed were measured using near-IR spectroscopy (Tango-R, Bruker Belgium N.V., Kontich, Belgium). Minerals and trace elements were analyzed by inductively coupled plasma atomic emission spectroscopy (ICP-AES; Avio 500, PerkinElmer, Waltham, MA, USA) according to the EN15510 method.

#### Zootechnical Performances and Fecal Consistency

Feed intake and body weight gain were weekly recorded throughout the entire experiment (*n* = 8 pens per treatment). The diarrhea status of the piglets was assessed visually, by a single observer, with fecal scoring every 2 days using a scale going from 1 to 3 (1 = hard or soft dry pellets; 2 = soft wet-shaped pellets; and 3 = unshaped soft pellets and watery feces). Each score was given per pen (*n* = 8 pens per treatment) and was converted into a weekly occurrence.

#### Sampling of Intestinal Tissues and Contents

One pig per pen, with an average pen body weight, was euthanized on post-weaning days 10–11, i.e., 5–6 days after receiving the experimental diets, and d31–32 post-weaning, when piglets were 31–32 and 52–53 days of age, respectively (*n* = 8 animals per treatment). The sampling points were organized in 2 consecutive days with a randomized scheme and an equal distribution of animals from each experimental group to be euthanized per day. Anesthesia was applied by an intramuscular injection of a mix of Xylazine (Dopharma, Raamsdonkveer, the Netherlands) and Zoletil 100 (Virbac, Barneveld, the Netherlands) resulting in doses of 4 mg kg^−1^ BW of xylazine, 2 mg kg^−1^ BW of zolazepam, and 2 mg kg^−1^ BW of tilamine. After anesthesia, piglets were euthanized with an intracardiac injection of sodium pentobarbital (0.2 ml kg^−1^ BW; Release®, ECUPHAR NV/SA, Oostkamp, Belgium) and were immediately exsanguinated by the severance of the carotid arteries and jugular veins.

Tissue samples were collected from the duodenum (about 15 cm after the pyloric junction), jejunum (middle section of the small intestine), and ileum (about 20 cm from the ileocecal valve), rinsed with a saline solution, and fixed in phosphate-buffered formalin (10%, pH 7.6) for 48 h prior to storage in 70% ethanol for histomorphometric measurements. Colonic tissues were rinsed with saline, snap-frozen, and stored at −80°C until the gene expression assay. Ileal, cecal, and colonic contents were collected. Ileal content was immediately stored on wet ice until viscosity measurements. Furthermore, ileal, cecal, and colonic contents were snap-frozen and stored at −80°C until further analyses of metabolites.

#### Viscosity Measurements

Soluble fibers are more extensively and rapidly fermented than insoluble polysaccharides due to their higher water-holding capacity, which increases the viscosity of the digested and allows bacteria to easily penetrate the matrix (36). Therefore, the viscosity of the ileal digesta was determined. Ileal digesta (*n* = 8 animals per treatment) collected on d31–32 were centrifuged for 15 min at 21,000 *g*, and the viscosity of a 500-μl supernatant was measured with a CP40 cone and a constant shear rate of 450 s^−1^ (Brookfield DV II+ viscometer; Brookfield, Middleboro, MA, USA). Viscosity measurements could not be achieved on d10–11 because of the lack of sufficient ileal content.

#### Histomorphology Metrics

Tissues from the duodenum and jejunum (*n* = 8 animals per treatment/sampling time) collected on d10–11 and d31–32, were embedded in paraffin wax, cut at a 5-μm thickness with a microtome using Thermo MX35 Ultra blades (Thermo Fisher Scientific, Waltham, MA, USA), and stained with Alcian Blue-Periodic Acid Schiff. Twenty well-oriented villus-crypt units were selected on each slide to determine villus height and width as well as crypt depth (μm) by 10-fold microscopy (Olympus Corporation, Tokyo, Japan). The number of acidic and neutral goblet cells (count per crypt), the density in total goblet cells (count per 100 μm of the crypt), the thickness of the *muscularis mucosae* and *tela submucosa*, and the *tunica muscularis* thickness (μm) were determined.

#### Gene Expression Assay in Colonic Tissue

Total RNA from colonic tissues (*n* = 8 animals per treatment/sampling time) collected on d10–11 and d31–32 was extracted using the ReliaPrep RNA Tissue Miniprep System (Promega Corporation, Madison, WI, USA) according to the protocol of the manufacturer. RNA concentration and quality were determined by Nanodrop dosage (Thermo Fisher Scientific, Waltham, MA, USA) and agarose gel (1%), respectively. The extracted RNA (75 ng) was converted into cDNA using the Reverse Transcription Master Mix (Fluidigm Corporation, South San Francisco, CA, USA). Samples were thereafter pre-amplified according to the PreAmp MasterMix instructions provided by the manufacturer (Fluidigm Corporation, South San Francisco, CA, USA), followed by an exonuclease I treatment (New England Biolabs, Ipswich, MA, USA).

High-throughput quantitative PCR (qPCR) was performed as previously described (35) with intron-spanning primer pairs (Supplementary Table 1) designed using Primer-BLAST (NCBI) and validated through agarose gel electrophoresis and through melting curves. High-throughput qPCR was performed in 48 × 48 dynamic array-integrated fluidic circuits (Fluidigm Corporation, South San Francisco, CA, USA) following the protocol: 60 s at 95°C, followed by 30 cycles (5 s at 96°C and 20 s at 60°C). Quantification cycles (*Cq*) were acquired using the Fluidigm real-time PCR analysis software 3.0.2 (Fluidigm Corporation, South San Francisco, CA, USA).

First, all housekeeping genes were evaluated, and the four most stable genes between treatments were determined by NormFinder (37). The selected four reference genes consisted of hypoxanthine phosphoribosyltransferase 1 (*HPRT1*), ribosomal protein L4 (*RPL4*), TATA-box binding protein (*TBP*), and tyrosine 3-monooxygenase/tryptophan 5-monooxygenase activation protein zeta (*YWHAZ*). For each target and housekeeping gene analyzed, the relative gene expression level was calculated using the Pfaffl method (38), and the geometrical mean of the relative expression of the four housekeeping genes was used to normalize all samples.

#### High-Performance Liquid Chromatography Metabolite Profiling of Ileal, Cecal, and Colonic Contents

Ileal, cecal, and colonic contents collected on d10–11 and d31–32 of the experiment (*n* = 8 animals per treatment/sampling time/intestinal segment) were six-fold diluted in ultrapure water prior to metabolite determination by high-performance liquid chromatography (HPLC). Intermediate metabolites (lactate, pyruvate, succinate, and formate), SCFAs (acetate, propionate, and butyrate), and branched-chain fatty acids (BCFAs; *i*-butyrate, *i*-valerate, and valerate) were analyzed by isocratic HPLC using an Alliance System e2695 (Waters Corporation, Milford, MA, USA) with an Aminex HPx-87H column (BioRad, Hercules, CA, USA) combined with a UV detector (210 nm), with H_2_SO_4_ (5 mM) as a mobile phase at an eluent flow of 0.6 ml min^−1^ and with a temperature of 60°C. Each peak was integrated using the Empower 3 software (Waters Corporation, Milford, MA, USA) and quantified using an external standard calibration. Although valerate is not a BCFA *per se*, the organic acid is usually classified as a metabolite from the proteolytic fermentation and is therefore included within the BCFA group. Intermediate metabolites and the sum of SCFAs were expressed in mg g^−1^ of fresh content. Acetate, propionate, butyrate, and BCFA amounts were expressed as a ratio (%) of the sum of SCFAs (39). As there were only differences in SCFA and BCFA in the colonic content, these samples were chosen for further metabolome analyses by gas chromatography-mass spectrometry (GC-MS) and liquid chromatography-mass spectrometry (LC-MS).

#### GC-MS Metabolomic Profiling of Colonic Contents

The GC-MS analysis was performed at the VIB metabolomics core Ghent (Ghent, Belgium). Samples were dissolved in 1 ml of methanol and divided into two aliquots of 400 μl each. For each sample, one of the aliquots was evaporated to dryness under vacuum, and the obtained residue was derivatized by adding 10 μl of pyridine and 50 μl of N-Methyl-N-(trimethylsilyl)-trifluoroacetamide (Sigma-Aldrich, Saint Louis, MO, USA). The GC-MS analysis was carried out using a 7,890B GC system equipped with a 7,693A Automatic Liquid Sampler and a 7,250 Accurate-Mass Quadrupole Time-of-Flight (QTOF) MS system (Agilent Technologies, Santa Clara, CA, USA). About 1 μl of the sample was injected in a split-less mode with an injector port set to 280°C. Separation was achieved with a VF-5ms column (30 m × 0.25 mm × 0.25 μm; Varian CP9013; Agilent Technologies, Santa Clara, CA, USA) with helium as a carrier gas at a constant flow of 1.2 ml/min. The oven was held at 80°C for 1 min, ramped to 280°C at 5°C/min, held at 280°C for 5 min, ramped to 320°C at 20°C/min, held at 320°C for 5 min, and finally cooled to 80°C at 50°C/min at the end of the run. The MSD transfer line was set to 280°C, and the electron ionization energy was 70 eV. Full EI-MS spectra were recorded between m/z 50–800 at a resolution of >25,000.

Data processing was done in MassHunter Profinder (Agilent Technologies, Santa Clara, CA, USA) and includes feature extraction, combined with a chromatographic alignment across multiple data files. The appearance of both false positive and false negative features is minimized by “binning” the features in a chromatographic time domain. The NIST 17 Mass Spectral Library was accessed to screen for the spectra that matched the compounds present in the GC-MS chromatogram.

#### LC-MS Metabolomics Profiling of Colonic Contents

The LC-MS analysis was performed at the VIB metabolomics core Ghent (Ghent, Belgium). Samples were dissolved in 1 ml of methanol and divided into two aliquots of 400 μl each. For each sample, one of the aliquots was evaporated to dryness under vacuum, and the obtained residue was reconstituted in cyclohexane/water (1:1 v/v) (Sigma-Aldrich, Saint Louis, MO, USA). The extract in the water phase was used for the following ultra-high-performance liquid chromatography (UHPLC) analysis. The latter was performed on an ACQUITY UPLC I-Class system (Waters Corporation, Milford, MA, USA) consisting of a binary pump, a vacuum degasser, an autosampler, and a column oven. Chromatographic separation was carried out on an ACQUITY UPLC BEH C18 (150 × 2.1 mm, 1.7 μm) column (Waters Corporation, Milford, MA, USA) at 40°C. A gradient of two buffers was used: buffer A (99:1:0.1 water:acetonitrile:formic acid, pH 3) and buffer B (99:1:0.1 acetonitrile:water:formic acid, pH 3), as follows: 99% A for 0.1 min decreased to 50% A in 30 min, decreased to 30% in 5 min, and decreased to 0% in 2 min. The flow rate was set to 0.35 ml min^−1^, and the injection volume was 10 μl. The UHPLC system was coupled to a Vion IMS QTOF hybrid mass spectrometer (Waters Corporation, Milford, MA, USA). The LockSpray ion source was operated in negative electrospray ionization (ESI) mode under the following specific conditions: capillary voltage, 2.5 kV; reference capillary voltage, 3 kV; cone voltage, 40 V; source offset, 50 V; source temperature, 120°C; desolvation gas temperature, 600°C; desolvation gas flow, 800 L h^−1^; and cone gas flow, 50 L h^−1^. Mass range was set from 50 to 1,000 Da. The collision energy for full HDMSe was set at 6 eV (low energy) and ramped from 20 to 70 eV (high energy), the intelligent data capture intensity threshold was set at 5. Nitrogen (greater than 99.5%) was employed as desolvation and cone gas. Leucin-enkephalin (250 pg μl^−1^ solubilized in water:acetonitrile 1:1 [v/v], with 0.1% formic acid) was used for the lock mass calibration, with scanning every 2 min at a scan time of 0.1 s.

Profile data were recorded through a UNIFI Scientific Information System (Waters Corporation, Milford, MA, USA). ESI interface was employed in both positive and negative modes. Data processing was performed with Progenesis QI software version 2.4 (Waters Corporation, Milford, MA, USA). The in-house Mass Spectral Library (PhytoComp) and external spectral libraries (MONA; https://mona.fiehnlab.ucdavis.edu/) were used to screen for the spectra that match the compounds present in the LC-MS chromatogram.

#### DNA Extraction, Sequencing, and Bioinformatics of Colonic Contents

DNA from colonic contents collected on d10–11 and d31–32 of the experiment (*n* = 8 animals per treatment/sampling time) was extracted using the QIAamp PowerFaecal Pro DNA kit (Qiagen, Hilden, Germany) following the instructions of the manufacturer. The DNA concentration and quality were determined by Nanodrop dosage (Thermo Fisher Scientific, Waltham, MA, USA) and agarose gel (1%), respectively. Following DNA extraction, 16S rRNA gene sequencing was performed by DNAVision (Gosselies, Belgium), using the Illumina MiSeq technologies (2 × 250 nt). The F-primer (5′-TCGTCGGCAGCGTCAGATGTGTATAAGAGACAGCCTACGGGNGGCWGC AG-3′) and the R-primer (5′-GTCTCGTGGGCTCGGAGATGTGTATAAGAGACAGGACTA CHVGGGTATCT AATCC-3′) were used to amplify the hypervariable regions V3-V4 according to the 16S Metagenomic Sequencing Library Preparation protocol from Illumina.

Quantitative Insights into Microbial Ecology (QIIME) software package (version 1.9.0) was used for operational taxonomic unit (OTU) clustering, with an identity cutoff of 97% by comparison to the Greengenes reference database 13.8. Beta diversity was measured using weighted UniFrac distance metrics with principal coordinate analysis (PCoA) using QIIME. The ADONIS method was used to determine whether communities differed significantly between groups of samples. Microbiota results were analyzed per time point using a Kruskall–Wallis test with the treatment as a fixed factor. The false discovery rate (FDR) correction was used to calculate the adjusted *p*-values. Microbial alpha diversity metrics, including Chao1, phylogenetic diversity whole tree, observed OTU, and Shannon indexes, were calculated using QIIME. Raw sequences have been uploaded to the European Nucleotide Archive database (project number PRJEB38284). Pearson's correlation coefficients between metabolites and microbiota communities were also calculated.

### Experiment 2

#### Animals

A second experiment was run in Gembloux Agro-Bio Tech, University of Liège, in Gembloux (Belgium) to determine intestinal permeability. All experimental procedures on piglets were in accordance with European and Belgian regulations concerning the care and use of animals for research purposes and were approved by the Animal Ethical Committee of Liège University, Belgium (protocol number: 1,860).

A total of 32 male Landrace × Piétrain piglets weaned at 21 days and with an average weight of 5.9 ± 0.6 kg (mean ± SD) were obtained from the Walloon Agricultural Research Center in Gembloux (Belgium). The animals were allocated to 16 pens of 2 littermates based on body weight. Piglets were housed in a temperature-controlled animal house with slatted floors and with a feeder and a drinking nipple per pen. Following the 5-day adaptation period with the commercial creep feed diet, piglets were assigned to one of the four treatments (*n* = 4 pens): a control diet, an IN diet at 0.2% (IN), and two CP diets (CP0.2/CP2%). Throughout the experiment, the pigs had *ad libitum* access to feed and water.

#### *In vivo* Intestinal Permeability

Intestinal permeability was assessed *in vivo* after 7 days of treatment with a sugar absorption test. Pigs (*n* = 8 per treatment) were initially fasted overnight and were subsequently administered with an oral dose of 5 ml kg^−1^ of sugar solution, containing D-xylose (100 mg kg^−1^; VWR International, Oud-Heverlee, Belgium) dissolved in water. One hour after oral administration, a 5-ml blood sample was collected from the jugular vein into gel and clot activator vacuum tubes (VWR International, Oud-Heverlee, Belgium). After centrifugation (10 min, 2,000 g at 4°C), serum samples were stored at −20°C until the analysis of serum concentration of D-xylose as a marker of intestinal absorptive capacities and mucosal integrity.

#### Determination of D-Xylose in Serum

Serum D-xylose was determined as described by Eberts et al. (40). Briefly, D-xylose standard solutions were prepared by dissolving D-xylose in saturated benzoic acid to reach 0, 50, 100, 200, 400, and 800 mg L^−1^ concentrations. The phloroglucinol color reagent solution was prepared with 0.5 g of phloroglucinol, 100 ml of glacial acetic acid, and 10 ml of concentrated hydrochloric acid (all from Sigma-Aldrich Co., St Louis, MO, USA).

The standard and serum samples (50 μl) were added to 5 ml of phloroglucinol color reagent solution, were heated at 100°C for 4 min, and were allowed to cool down in a water bath at room temperature. The absorbance was determined at 570 nm in a spectrophotometer (VICTOR plate reader, PerkinElmer, Waltham, MA, USA). The standard solution of 0 mg L^−1^ D-xylose was considered as blank, and xylose-free serum was used to obtain the net absorptive concentrations.

### Statistical Analyses

Zootechnical performance was analyzed with mixed procedures, the treatment being a fixed factor, the pen being a random factor, and using the initial body weight as a covariate. Fecal scoring was also analyzed using a mixed procedure with the treatment as a fixed factor and the pen as a random factor. Viscosity measurements and serum xylose concentrations were subjected to generalized linear model (GLM) procedures using one fixed criterion of classification (treatment). Microbiota and metabolomic results and gene expression data were analyzed per time point, using the treatment as a fixed factor in the GLM procedure. The FDR correction was used to calculate the adjusted *p*-values for the gene expression and microbiota and metabolomic results (41). The histomorphometrical data were analyzed per time point, using the treatment as a fixed factor and the slaughtering weight as a covariate in the GLM procedure. Gene expression and histomorphometrical data were analyzed per time point as we chose to focus on the treatment effect rather than the age effect, which was not considered as the main aim of this research. The comparison of means was evaluated by *post hoc* Tukey's multiple comparison honestly significant difference (HSD). For the metabolomic data, ANOVA, heatmaps, and PCA plots were generated using the online tool MetaboAnalyst 4.0. The *P*-values <0.05, *p* < 0.01, and *p* < 0.001 were considered as statistically significant, highly significant, and very highly significant. All statistical analyses were performed using SAS 9.4 (SAS Institute, Inc., Cary, NC, USA).

## Results

### Experiment 1

#### Zootechnical Performances

**Figure 1:**
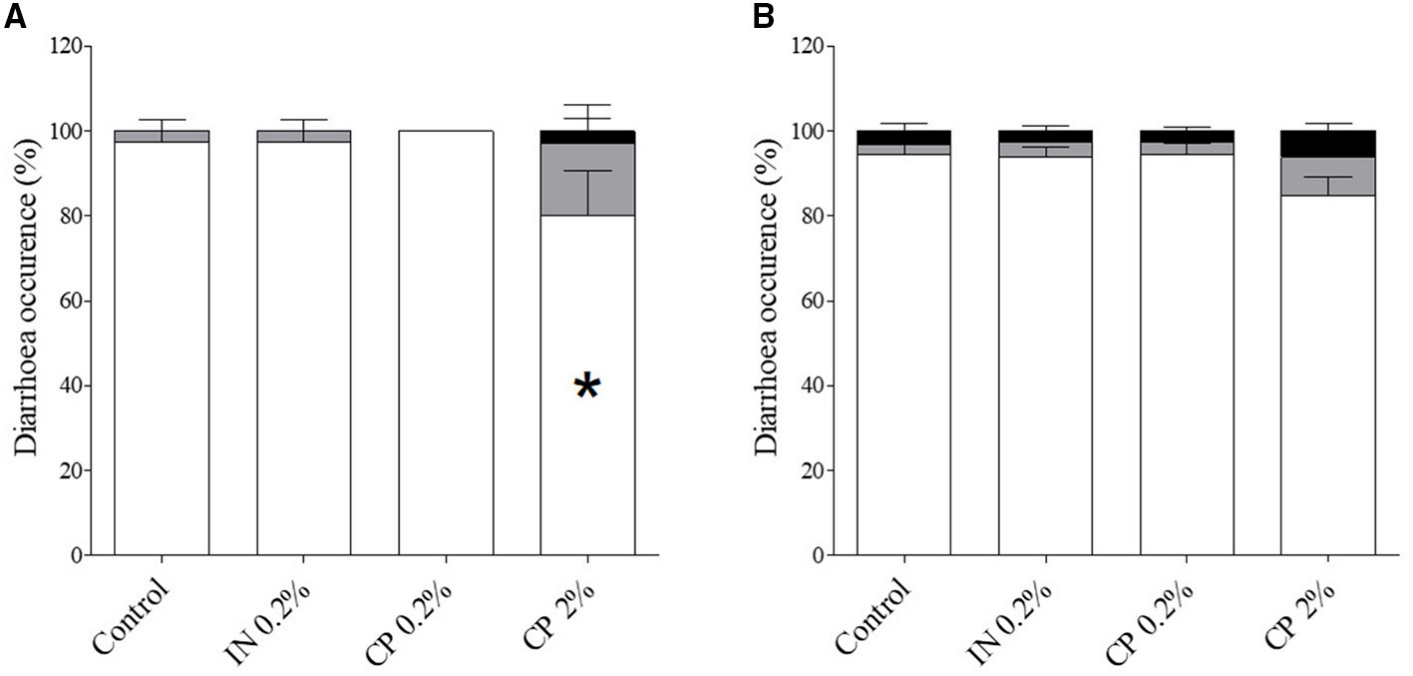
Diarrhea occurrence in pigs after feeding inulin (IN) (IN0.2%) and citrus pulp (CP) (CP0.2% and CP2%) from post-weaning day 5, after 2 weeks **(A)** and for the entire 4-week experiment **(B)**. 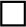 *Score 1* (hard or soft dry pellets); 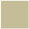 *Score 2* (soft wet-shaped pellets); 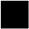 Score 3 (unshaped soft pellets and watery feces). Mean values (*n* = 8 pens) ± SEM. Symbols (*) within bars indicate significant differences between groups (*p* <0.05).

Weekly body weight gain and feed intake remained unaffected by diets (*p* > 0.05). Piglets fed with CP2% displayed a significantly higher feed conversion ratio (1.66 ± 0.05) in comparison to their control counterparts (1.45 ± 0.06) after 1 week of treatment (*p* < 0.05), which was no longer observed during the 3 following weeks and for the entire experimental period (Supplementary Table 2). Piglet receiving CP2% demonstrated significantly lower proportions of score 1 feces, dry pellets, during the 2nd week of treatment (Figure 1A). No other significant differences were observed for weeks 1, 3, and 4 (data not shown) or for the entire experimental period (Figure 1B).

#### The Viscosity of the Ileal Digesta

The CP2% diet (2.69 ± 0.36 cP) induced a significant rise in ileal chyme viscosity in comparison with control (1.48 ± 0.06 cP) and CP0.2% (1.48 ± 0.09 cP) treatments on d31–32 (*p* <0.0001), while IN-treated pigs (2.03 ± 0.03 cP) demonstrated intermediate values.

#### Small Intestinal Histomorphology

At d10–11, histological measurements showed a significantly reduced crypt depth with increased VH:CD ratio due to the treatment with CP and IN in all the studied segments of the intestine, including the duodenum, jejunum, and ileum (Table 2). The measurements at d31–32 showed a decrease in crypt depth in the duodenum (*p* < 0.05) and ileum (0.05 < *p* <0.1) and a significant increase of VH:CD ratio due to the treatment in the duodenum, jejunum, and ileum (*p* < 0.05).

**Table 2:** Histomorphometrical measurements in the duodenum, jejunum, and ileum intestinal segments after feeding inulin (IN) (IN0.2%) and citrus pulp (CP) (CP0.2% and CP2%) from post-weaning day 5 onward, at sampling times on d10–11 and d31–32 post-weaning.

		**Control**	**IN0.2%**	**CP0.2%**	**CP2%**	***P-*value**
**d10–11**						
Villus height (μm)	Duodenum	436 ± 11	479 ± 19	430 ± 15	443 ± 14	0.1226
	Jejunum	448 ± 11	484 ± 17	504 ± 19	479 ± 17	0.13
	Ileum	388 ± 9	413 ± 13	411 ± 10	401 ± 11	0.3518
Villus width (μm)	Duodenum	127 ± 6	124 ± 7	117 ± 2	123 ± 6	0.6014
	Jejunum	167 ± 47	118 ± 4	115 ± 3	111 ± 3	0.3321
	Ileum	113 ± 1	108 ± 4	108 ± 3	111 ± 3	0.5098
Crypt depth (μm)	Duodenum	378^a^±13	312^b^±9	306^b^±9	320^b^±11	**0.0001**
	Jejunum	285^a^±7	251^b^±7	251^b^±5	251^b^±7	**0.0012**
	Ileum	245^a^±8	223^a^^b^±7	216^b^±4	213^b^±5	**0.0047**
VH:CD ratio^*^	Duodenum	1.17^b^±0.02	1.57^a^±0.07	1.44^a^±0.07	1.42^a^±0.06	**0.0002**
	Jejunum	1.60^b^±0.03	1.96^a^±0.06	2.06^a^±0.1	1.94^a^±0.05	**0.0002**
	Ileum	245^a^±8	223^a^^b^±7	216^b^±4	213^b^±5	**0.0047**
Neutral goblet cell count (count per crypt)	Duodenum	3.5 ± 2.5	0.6 ± 0.2	0.9 ± 2	1.3 ± 0.3	0.3625
	Jejunum	1.7 ± 0.5	1.1 ± 0.3	0.6 ± 0.2	1.2 ± 0.4	0.2862
	Ileum	0.9 ± 0.2	0.8 ± 0.2	0.7 ± 0.2	0.5 ± 0.1	0.3562
Acidic goblet cell count (count per crypt)	Duodenum	18.6^a^±1.5	10.7^b^±1.1	12.5^b^±1	12.7^b^±1.6	**0.0013**
	Jejunum	11.8 ± 1.1	9.8 ± 0.9	10.3 ± 0.9	8.9 ± 0.9	0.2134
	Ileum	15.9^a^±0.5	11.9^b^±0.8	10.8^b^±1.2	11.4^b^±1	**0.0019**
Total goblet cell density (count per 100 μm crypt)	Duodenum	5.7 ± 0.8	3.6 ± 0.3	4.4 ± 0.3	4.3 ± 0.5	0.0576
	Jejunum	4.8 ± 0.4	4.3 ± 0.3	4.4 ± 0.4	4 ± 0.4	0.5841
	Ileum	6.9^a^±0.2	5.7^a^^b^±0.3	5.3^b^±0.5	5.6^a^^b^±0.4	**0.0255**
**d31–32**						
Villus height (μm)	Duodenum	563 ± 20	604 ± 23	575 ± 25	606 ± 24	0.4808
	Jejunum	540 ± 17	529 ± 11	572 ± 19	545 ± 19	0.3334
	Ileum	417 ± 13	460 ± 25	487 ± 17	456 ± 25	0.1519
Villus width (μm)	Duodenum	145 ± 5	145 ± 12	137 ± 3	141 ± 5	0.856
	Jejunum	133 ± 4	123 ± 3	127 ± 2	123 ± 2	0.085
	Ileum	125 ± 3	128 ± 3	128 ± 3	131 ± 7	0.7975
Crypt depth (μm)	Duodenum	375^a^±8	334^b^±13	326^b^±6	338^a^^b^±10	**0.0058**
	Jejunum	313 ± 8	275 ± 7	289 ± 8	304 ± 33	0.4634
	Ileum	248 ± 4	231 ± 7	241 ± 7	224 ± 8	0.0956
VH:CD ratio^*^	Duodenum	1.53^b^±0.03	1.86^a^±0.05	1.80^a^±0.07	1.83^a^±0.06	**0.0009**
	Jejunum	1.75^b^±0.04	1.96^a^^b^±0.05	2.01^a^±0.08	2.03^a^±0.06	**0.0083**
	Ileum	1.71^b^±0.06	2.02^a^±0.07	2.06^a^±0.08	2.07^a^±0.08	**0.0046**
Neutral goblet cell count (count per crypt)	Duodenum	1.1 ± 0.3	0.8 ± 0.2	0.8 ± 0.2	0.6 ± 0.2	0.4287
	Jejunum	0.1 ± 0.1	0.1 ± 0.1	0.1 ± 0.1	0.1 ± 0.1	0.8297
	Ileum	0.4 ± 0.1	0.8 ± 0.3	0.7 ± 0.2	0.6 ± 0.2	0.438
Acidic goblet cell count (count per crypt)	Duodenum	16.6 ± 1.6	15.9 ± 1.8	13.3 ± 1.2	14.4 ± 1.6	0.438
	Jejunum	13.8^a^±1.2	10.2^b^±0.6	11.2^a^^b^±0.8	10.3^b^±0.9	**0.0285**
	Ileum	11.8 ± 1	10.5 ± 0.7	10.8 ± 0.9	11.4 ± 1.2	0.7514
Total goblet cell density (count per 100 μm crypt)	Duodenum	4.7 ± 0.5	5 ± 0.5	4.3 ± 0.4	4.5 ± 0.5	0.7507
	Jejunum	4.4 ± 0.3	3.8 ± 0.3	3.9 ± 0.3	3.6 ± 0.4	0.3624
	Ileum	4.9 ± 0.4	4.9 ± 0.2	4.7 ± 0.3	5.3 ± 0.5	0.6448

* *VH:CD, villus height to crypt depth ratio*.

*Mean values ± SEM (n = 8 animals)*.

a, b *Mean values within a row with unlike superscript letters are significantly different (p < 0.05)*.

*The P-values < 0.05, p < 0.01, and p <0.001 were considered as statistically significant, highly significant, and very highly significant*.

Inulin-treated pigs displayed the thickest and the thinnest duodenal *muscularis mucosae* and *tela submucosa* layers on d10–11 and d31–32, respectively, reaching statistical trends (*p* < 0.1; Supplementary Table 3). No further differences were observed in other histomorphometrical measurements.

Acidic goblet cells showed a significant decrease in counts due to the treatment at days 10–11 in the duodenum and ileum (*p* < 0.05) (Table 2). Total goblet cell counts were also significantly affected by the treatment in the ileum section of the intestine (*p* < 0.05), while the duodenum showed a tendency. At the sampling times of 31–32 days, the acidic goblet cell count was significantly reduced due to the treatment in the jejunum section (*p* < 0.05), while it remained unaffected in the other sections.

#### Gene Expression in Colonic Tissue

**Figure 2:**
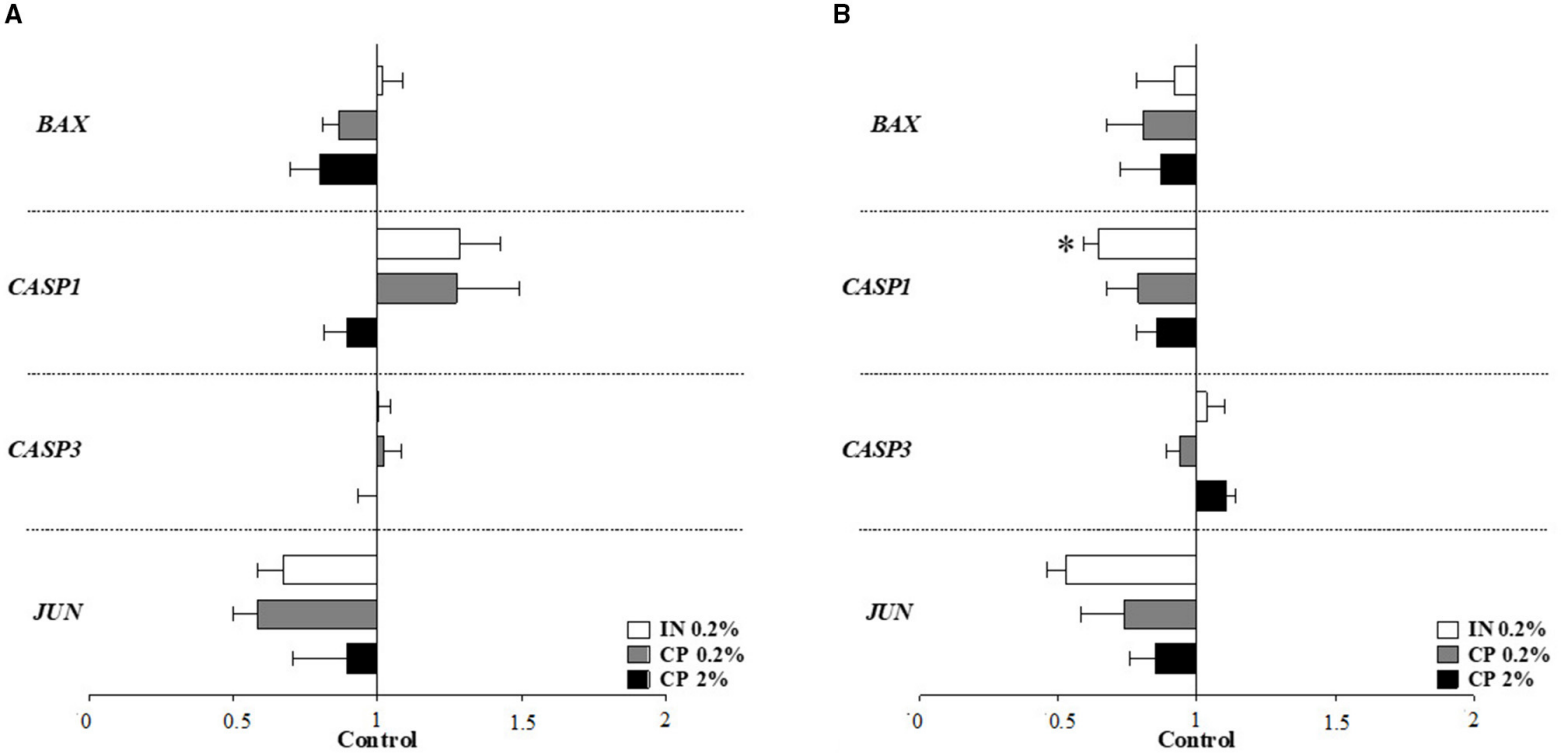
Apoptosis-related target gene expression in the colonic tissue of pigs after feeding IN (IN0.2%) and CP (CP0.2% and CP2%) from post-weaning day 5, at sampling times on d10–11 **(A)** and d31–32 **(B)** post-weaning. BAX, BCL2-associated X protein; CASP, caspase; JUN, AP-1 transcription factor subunit. Figures display the % of the difference in comparison to the control treatment, considered as 1. Mean values (*n* = 8 animals) ± SEM. Symbol (*) denotes a significant difference between the treatments and the control (*p* < 0.05).

**Figure 3:**
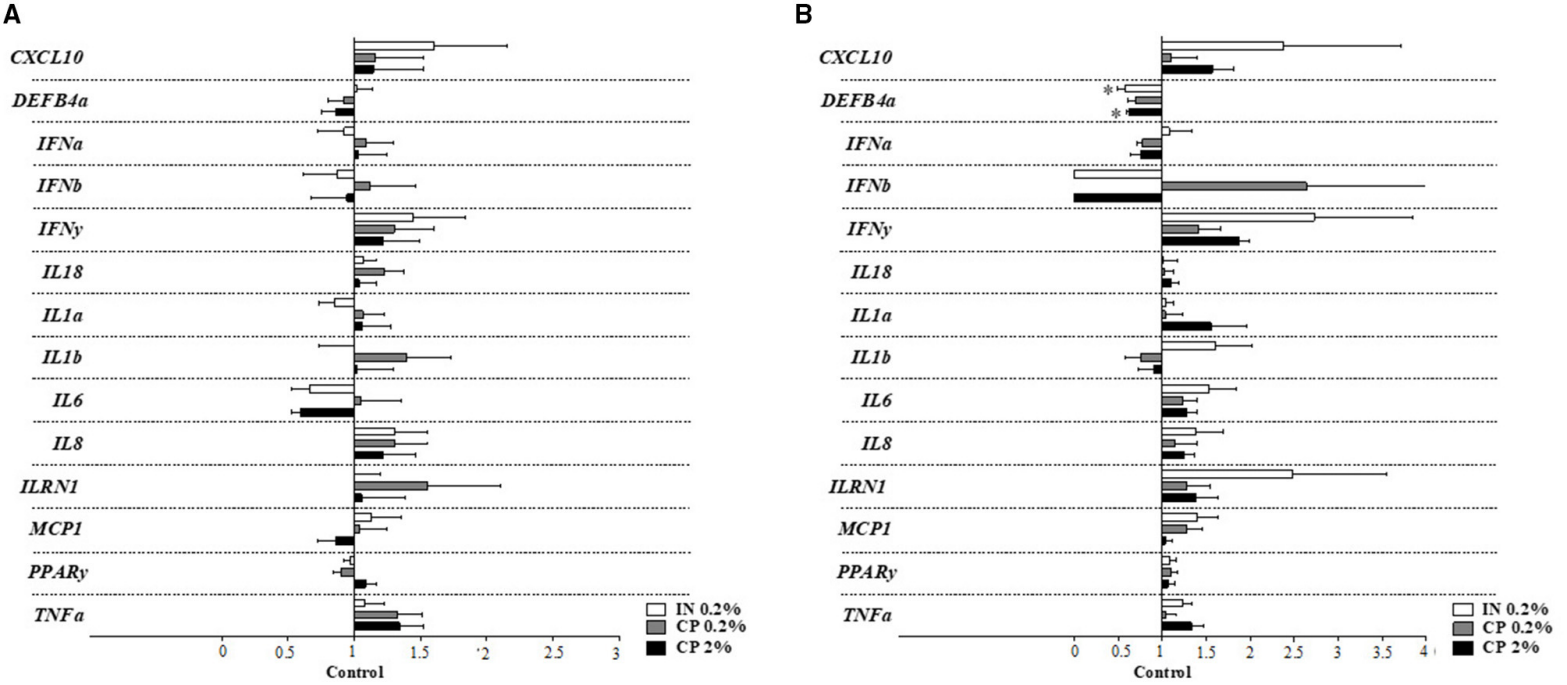
Inflammatory target gene expression in the colonic tissue of pigs after feeding IN (IN0.2%) and CP (CP0.2% and CP2%) from post-weaning day 5, at sampling times on d10–11 **(A)** and d31–32 **(B)** post-weaning. CXCL10, C-X-C motif chemokine 10; DEFβ, defensin beta; IFN, interferon; IL, interleukin; ILRN1, interleukin-1 receptor antagonist; MCP-1, monocyte chemo attractant protein 1; PPARγ, peroxisome proliferator-activated receptor gamma; TNFα, tumor necrosis factor alpha. Figures display the % of the difference in comparison to the control treatment, considered as 1. Mean values (*n* = 8 animals) ± SEM. Symbol (*) denotes a significant difference between the treatments and the control (*p* < 0.05).

**Figure 4:**
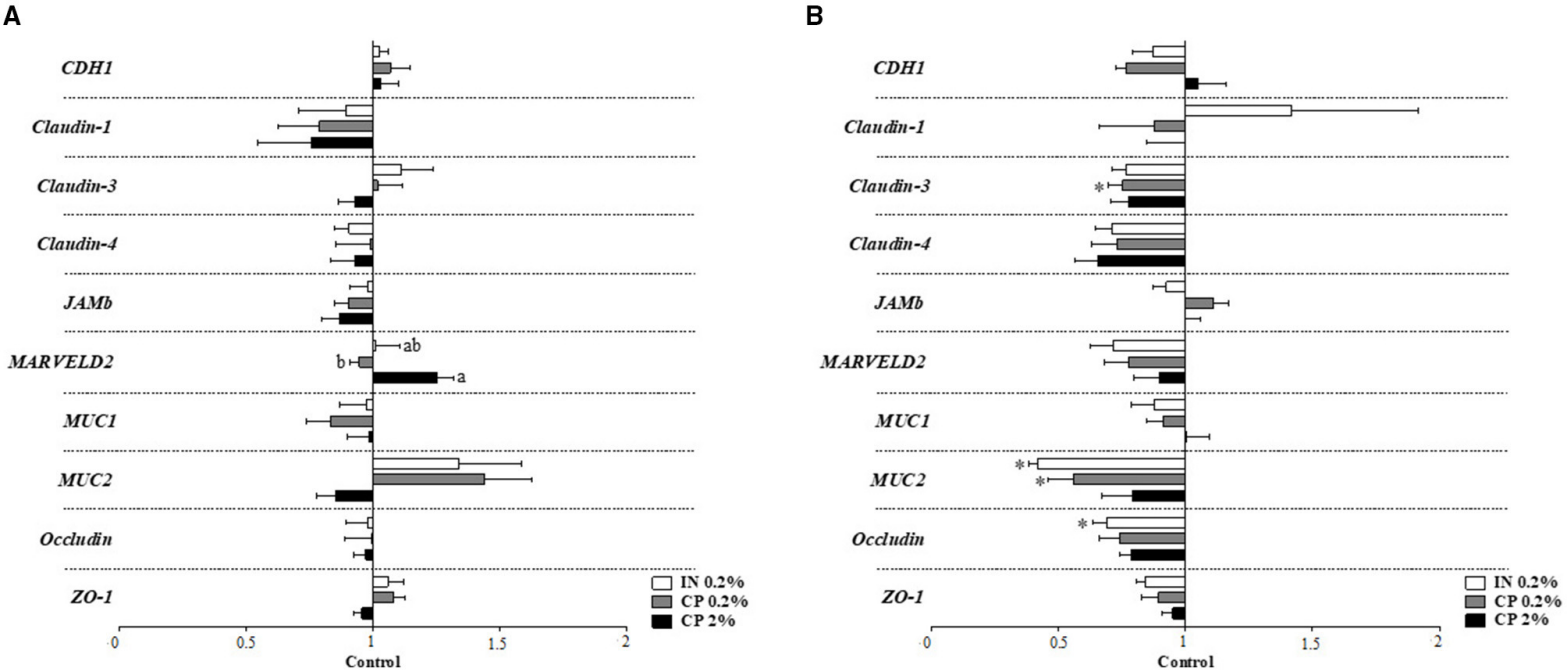
Barrier-integrity target gene expression in the colonic tissue of pigs after feeding IN (IN0.2%) and CP (CP0.2% and CP2%) from post-weaning day 5, at sampling times on d10–11 **(A)** and d31–32 **(B)** post-weaning. CDH1, E-cadherin; JAM, junctional adhesion molecule; MARVELD2, tricellulin; MUC, mucin; ZO-1, zonula occludens-1. Figures display the % of the difference in comparison to the control treatment, considered as 1. Mean values (*n* = 8 animals) ± SEM. ^a, b^Different superscript letters denote a significant difference between IN, CP0.2%, and CP2% (*p* < 0.05). Symbol (*) denotes a significant difference between the treatments and the control (*p* < 0.05).

Apoptosis-related genes in a colonic tissue (Figure 2) showed downregulation of caspase 1 (*CASP1*) in the IN treatment compared to control pigs on d31–32 (Figure 2B). None of the 11 genes involved in signaling pathways of inflammation were altered following IN or CP supplementation, neither on d10–11 nor on d31–32, as seen in Supplementary Figure 1 (*p* > 0.05). The inflammation target gene (Figure 3) β-defensin 2 expression was downregulated in the colonic tissue of CP2%- and IN-fed piglets in comparison to control piglets on d31–32 (Figure 3B). Barrier integrity genes (Figure 4) showed an upregulation of tricellulin (*MARVELD2*) on d10–11 in CP2%-treated pigs, which was significantly different from that in the CP0.2% treatment (Figure 4A). On d31–32, claudin-3 and mucin 2 (*MUC2*) mRNA levels were lower with CP 0.2% in comparison to the control diet. At the same time, IN-fed pigs showed decreased expressions of *MUC2* and occludin compared to their control counterparts (Figure 4B). The FDR correction did not reveal any difference between diets for any of the genes.

#### SCFA and BCFA Profiles in the Ileal, Cecal, and Colonic Contents

The ileal and cecal SCFA and BCFA profiles remained unaffected by treatments, regardless of the sampling time (Supplementary Table 4). On d10–11, piglets receiving the CP2% diet had significantly lower colonic BCFA proportions in comparison to their control counterparts (*p* < 0.01; Table 3). At that time, significantly higher colonic acetate ratios were found with the CP2% treatment in comparison to the other treatments (*p* < 0.01; Table 3). At the same time, colonic lactate levels significantly increased following IN supplementation in comparison to CP0.2% and CP2% treatments (*p* < 0.05; Table 3). On d31–32, the CP2% diet tended to induce lower colonic propionate proportions, reaching a statistical trend (*p* < 0.1; Table 3).

**Table 3:** Metabolite concentrations in the colonic content of piglets after feeding IN (IN0.2%) and CP (CP0.2% and CP2%) from post-weaning day 5 onward, at sampling times on d10–11 and d31–32 post-weaning.

**Sampling time**	**Treatment**	**Lactate (mg g^**−1**^ OM)**	**Pyruvate (mg g^**−1**^ OM)**	**Succinate (mg g^**−1**^ OM)**	**Formate (mg g^**−1**^ OM)**	**SCFAs (mg g^**−1**^ OM)**	**Acetate (%)**	**Propionate (%)**	**Butyrate (%)**	**BCFAs (%)**
d10–11	Control	0.3^a^^b^ ± 0.11	0.01 ± 0.01	0.02 ± 0.01	0 ± 0	8.33 ± 0.4	49.23^b^ ± 1.44	30.09 ± 1.52	17.01 ± 1.35	3.67^a^ ± 0.81
	IN0.2%	0.72^a^ ± 0.21	0.01 ± 0	0.03 ± 0.02	0 ± 0	7.85 ± 0.29	49.58^b^ ± 1.26	29.83 ± 0.76	18.02 ± 1.21	2.56^a^^b^ ± 0.7
	CP0.2%	0.18^b^ ± 0.07	0 ± 0	0 ± 0	0 ± 0	8.6 ± 0.22	49.13^b^ ± 1.3	31.38 ± 1.51	17.57 ± 1.53	1.92^a^^b^ ± 0.42
	CP2%	0.22^b^ ± 0.08	0.01 ± 0.01	0.01 ± 0	0 ± 0	8.31 ± 0.38	56.20^a^ ± 1.48	28.63 ± 0.78	14.53 ± 0.96	0.64^b^ ± 0.26
	*P*-value treatment	0.0229	0.4509	0.2343	0.4074	0.4641	**0.0022**	0.4634	0.2411	**0.0098**
d31–32	Control	0.28 ± 0.15	0 ± 0	0 ± 0	0 ± 0	7.92 ± 0.44	48.17 ± 1.3	29.47 ± 1.5	20.54 ± 1.29	1.82 ± 0.75
	IN0.2%	0.51 ± 0.45	0.01 ± 0.01	0 ± 0	0 ± 0	7.67 ± 0.49	48.99 ± 1.95	29.93 ± 1.12	20.88 ± 1.76	0.2 ± 0.17
	CP0.2%	0.16 ± 0.1	0 ± 0	0 ± 0	0 ± 0	8.42 ± 0.43	48.01 ± 1.45	26.38 ± 0.86	23.9 ± 1.07	1.72 ± 1.02
	CP2%	0.29 ± 0.09	0.01 ± 0.01	0 ± 0	0 ± 0	7.53 ± 0.44	52.59 ± 2.75	25.04 ± 2.3	20.58 ± 0.91	1.79 ± 0.92
	*P*-value treatment	0.7929	0.3848	0.7354	ND	0.5288	0.3241	0.0927	0.2198	0.4079

*ND, Not determined*.

*SCFAs = total amount of short-chain fatty acids (acetic + propionic + i-butyric + butyric + i-valeric + valeric acids; expressed as mg g^−1^ OM); acetic, propionic, and butyric acid proportions (expressed as % of total SCFAs)*.

*BCFAs = branched-chain fatty acid proportions (i-butyric + i-valeric + valeric acids scaled to SCFAs, expressed as %)*.

*Mean values (n = 8 animals) ± SEM*.

a, b *Mean values within a column with unlike superscript letters are significantly different (p < 0.05)*.

*The P-values < 0.05, p < 0.01, and p < 0.001 were considered as statistically significant, highly significant, and very highly significant*.

#### Metabolomic Profile in the Colonic Content

**Figure 5:**
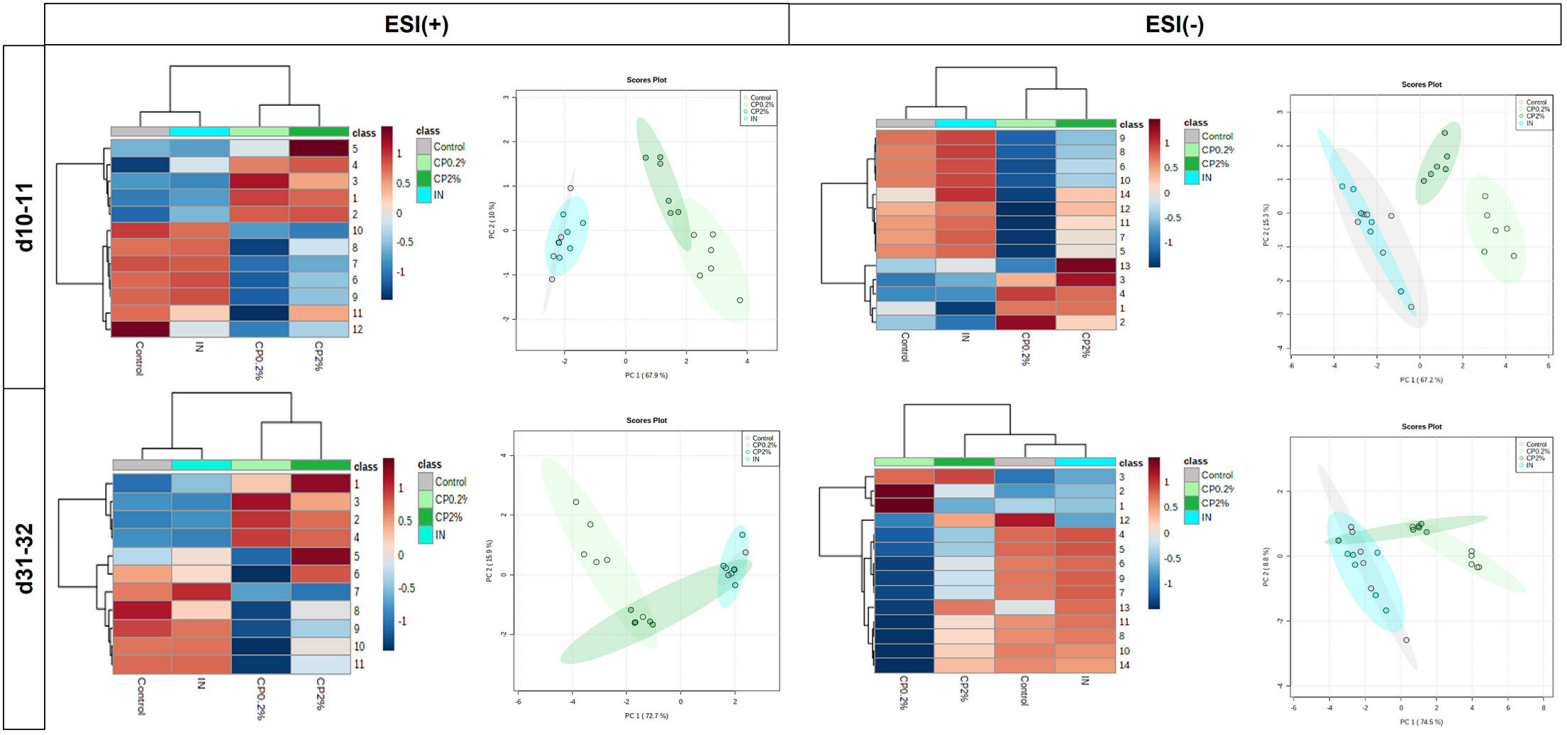
Heatmap (left) and PCA plots (right) of metabolomic analysis in the colon after feeding a diet supplemented with IN (IN0.2%) and CP (CP0.2% and CP2%) from post-weaning day 5 onward, at sampling times on d10–11 (top) and d31–32 (bottom) post-weaning. The results show the top most significant compound ions (*p* < 0.05) obtained from an analysis by liquid chromatography-mass spectrometry (LC-MS) (positive “+” and negative “–”; top left and right, respectively). Numbers refer to the labels used in Table 4 presenting the different compounds. The groups compared were control, IN, and CP (CP0.2% and CP2%). Each colored cell on the heatmap corresponds to the average concentration of the compound (row) per treatment group (column; *n* = 6 per group).

The top significant features obtained with the LC-MS results are shown in Table 4 and can be visualized in Figure 5. The ESI+ list showed 12 features at d10–11 and 11 features at d31–32. The ESI– list showed 14 features at d10–11 and 14 at d31–32, which mostly include flavonoids such as hesperidin, neohesperidin, naritin, and naringenin.

**Table 4:** Liquid chromatography-mass spectrometry (LC-MS) characterization of the main features using electrospray ionization (ESI) + and ESI – in the colonic content of piglets after feeding IN (IN0.2%) and CP (CP0.2% and CP2%) from post-weaning day 5 onward, at sampling times on d10–11 and d31–32 post-weaning. For the top compound ions, retention time (min) is shown, as well as the *p*-value and false discovery rate (FDR) for the differences between groups and the best identification hit.

**Sampling time**	**ESI**	**Label**	**Retenion time**	**Mass-to-charge ratio**	***p*-value**	**FDR**	**Best hit**
d10–11	Pos	1	13.41	611.1972 m/z	3.15E-18	3.76E-15	Hesperidin
		2	19.51	457.2220 m /z	4.00E-17	2.55E-14	Unknown
		3	22.26	517.2432 m/z	1.61E-17	1.24E-14	Unknown
		4	24.21	439.2114 m/z	7.57E-19	1.75E-15	Glycocholic acid
		5	27.46	817.4587 m/z	6.72E-16	2.83E-13	Unknown
		6	12.34	273.0755 m/z	9.01E-15	2.32E-12	Naringenin
		7	24.66	403.1384 m/z	8.80E-18	8.16E-15	Unknown
		8	7.41	718.3002 m/z	1.78E-15	6.37E-13	Unknown
		9	8.03	615.2906 m/z	3.10E-14	6.84E-12	Unknown
		10	19.04	475.2323 m/z	6.90E-15	1.88E-12	Unknown
		11	19.39	473.2165 m/z	5.66E-15	1.64E-12	Unknown
		12	22.66	373.1279 m/z	9.89E-15	2.41E-12	Tangeritin
	Neg	1	13.32	609.1822 m/z	4.95E-14	1.34E-11	Hesperedin
		2	22.16	515.2283 m/z	5.55E-13	1.09E-10	Unknown
		3	16.86	593.1871 m/z	7.70E-17	5.55E-14	Isosakuranetin-7-O-rutinoside
		4	24.03	531.2233 m/z	4.24E-17	4.58E-14	Taurocholic/ursodeoxycholic acid
		5	7.34	716.2849 m/z	4.48E-15	1.61E-12	Frangulin A
		6	15.67	297.1330 m/z	7.23E-17	5.55E-14	Linarin
		7	19.39	646.1340 m/z	4.16E-14	1.20E-11	[DAla2] Leu-Enkephalin
		8	13.34	301.0699 m/z	3.69E-16	1.77E-13	Hesperidin
		9	12.09	915.2767 m/z	1.67E-12	3.00E-10	Neohesperidin
		10	9.12	787.2300 m/z	2.55E-16	1.39E-13	Naringenin-7-O-glucoside
		11	12.26	678.0876 m/z	1.17E-12	2.19E-10	Nariturin
		12	27.37	839.4668 m/z	1.75E-15	6.89E-13	Unknown
		13	13.59	359.0763 m/z	2.08E-12	3.59E-10	Gossypetin 3,3',8-trimethylether/4',5,7-Trihydroxy 3,6,8-trimethoxyflavone
		14	15.92	699.2222 m/z	7.96E-14	1.91E-11	Unknown
d31–32	pos	1	27.46	817.4587 m/z	7.38E-19	9.96E-16	Unknown
		2	19.51	457.2220 m/z	3.69E-18	2.14E-15	Unknown
		3	22.26	517.2432 m/z	1.26E-21	2.92E-18	Unknown
		4	24.21	439.2114 m/z	3.24E-18	2.14E-15	Glycocholic acid
		5	15.4	345.0967 m/z	2.73E-15	7.02E-13	Unknown
		6	18.78	735.3225 m/z	5.17E-16	2.00E-13	Unknown
		7	19.04	475.2323 m/z	2.18E-15	5.94E-13	Unknown
		8	24.66	403.1384 m/z	1.98E-16	9.16E-14	Unknown
		9	19.39	473.2165 m/z	1.60E-15	4.92E-13	Unknown
		10	16.03	701.2369 m/z	2.46E-16	1.03E-13	Unknown
		11	7.11	647.2267 m/z	1.31E-15	4.34E-13	Unknown
	neg	1	24.03	531.2233 m/z	1.31E-15	7.07E-13	Taurocholic/ursodeoxycholic acid
		2	13.32	609.1822 m/z	1.71E-14	4.93E-12	Hesperedin
		3	16.86	593.1871 m/z	1.60E-15	7.66E-13	Isosakuranetin-7-O-rutinoside
		4	13.34	301.0699 m/z	1.95E-16	1.41E-13	Hesperidin
		5	15.67	297.1330 m/z	1.93E-21	8.34E-18	Linarin
		6	12.26	678.0876 m/z	8.28E-14	1.79E-11	Nariturin
		7	9.12	787.2300 m/z	1.60E-14	4.93E-12	Naringenin-7-O-glucoside
		8	20.93	517.4578 m/z	1.09E-15	6.72E-13	Unknown
		9	27.37	839.4668 m/z	2.28E-15	9.86E-13	Unknown
		10	18.66	711.3234 m/z	1.71E-16	1.41E-13	18-Hydroxy-5Z,8Z,11Z,14Z,16E-eicosapentaenoic acid
		11	19.39	646.1340 m/z	8.75E-15	2.91E-12	[DAla2] Leu-Enkephalin
		12	15.92	699.2222 m/z	5.94E-14	1.36E-11	Unknown
		13	12.09	915.2767 m/z	1.53E-16	1.41E-13	Neohesperidin
		14	9.82	718.3000 m/z	3.68E-14	9.94E-12	Unknown

Moreover, LC-MS resulted in an ESI– list containing 89 significant feature ions between the treatments at d10–11, while at d31–32 there were 100 features significantly different at an FDR < 0.05 and *p*-value < 0.05 (data not shown). Eighty-three features were in common between both time points. For an ESI+ list, at d10–11, there were 114 features significantly different, while at d31–32, 127 features were significantly different between treatments, at an FDR < 0.05 and p-value < 0.05. For this list, 104 features were in common between both time points (data not shown).

For all the colonic content samples analyzed, GC-MS resulted in the detection of 329 compounds. The list of the top 10 identified compounds and heatmaps obtained at d10–11 and d31–32 that had a p-value < 0.05 for the treatment effect are presented in Table 5 and visualized as (Supplementary Figure 2). None of them were significantly different between treatments after the FDR correction. Nonetheless, some of these identified compounds, such as octodecenoic acid, indole-acetic acid (IAA), and pipecolic acid, were interesting.

**Table 5:** Gas chromatography-mass spectrometry (GC-MS) characterization of the main features in the colonic content of piglets after feeding IN (IN0.2%) and CP (CP0.2% and CP2%) from post-weaning day 5, at sampling times on d10–11 and d31–32 post-weaning.

**Sampling time**	**Label**	**Compound**	***p*-value**	**FDR**	**Library (NIST) Match**	**Score**
d10–11	1	Compound262_33.36	0.010469	0.98931	Unknown	–
	2	Compound260_33.25	0.015944	0.98931	13-Trimethylsilyloxy-9-octadecenoic acid, methyl ester	77.09
	3	Compound235_30.07	0.010681	0.98931	13-Octadecenoic acid, methyl ester	82.93
	4	Compound60_14.84	0.045133	0.98931	Unknown	–
	5	Compound32_12.3	0.017057	0.98931	Unknown	–
	6	Compound79_17.35	0.049825	0.98931	Unknown	–
	7	Compound232_29.86	0.040642	0.98931	Unknown	–
	8	Compound55_14.62	0.01473	0.98931	Unknown	–
	9	Compound205_27.21	0.031758	0.98931	3-indoleacetic acid, 2TMS derivative	80.88
d31–32	1	Compound23_11.34	0.011181	0.69467	Unknown	–
	2	Compound235_30.07	0.044674	0.96277	13-Octadecenoic acid, methyl ester	83.2
	3	Compound190_26.05	0.0054548	0.59639	Unknown	–
	4	Compound303_41.41	0.00037716	0.12371	Unknown	–
	5	Compound57_14.66	0.0046701	0.59639	Unknown	–
	6	Compound153_23.34	0.012707	0.69467	Methyl-galactoside (1S, 2S, 3S, 4R, 5R)-, 4TMS derivative	83.41
	7	Compound296_39.78	0.031652	0.96277	Lactose, 8TMS derivative	83.61
	8	Compound50_13.85	0.010807	0.69467	Pipecolic acid, 2TMS derivative	75.73
	9	Compound327_49.72	0.034089	0.96277	Unknown	–

*For the top compound ions, retention time (min) is shown, as well as the p-value and FDR for the differences between groups and the best identification hit*.

#### Microbiota in the Colonic Content

On d10–11, the observed OTU and phylogenetic diversity whole tree indexes were significantly impacted by treatments, whereas no significant differences were found for the Chao1 and Shannon indexes (Table 6), neither on d10–11 nor on d31–32. On d10–11, according to both the observed OTU and phylogenetic diversity whole tree indexes, higher alpha diversity was found with the IN treatment in comparison to the CP0.2% treatment (*p* < 0.05). Concerning the observed OTU index, the IN treatment was also significantly higher than the control treatment (*p* < 0.05).

**Table 6:** Microbial alpha diversity after feeding IN (IN0.2%) and CP (CP0.2% and CP2%) from post-weaning day 5 onward, at sampling times on d10–11 and d31–32 post-weaning.

**Indexes**	**Sampling time**	**Control**	**IN0.2%**	**CP0.2%**	**CP2%**	***P*-value treatment**
Chao1	d10–11	1,069.52 ± 115.20	1,241.23 ± 84.18	1,122.04 ± 92.40	1,204.28 ± 156.77	NS
	d31–32	1,202.98 ± 121.84	1,267.39 ± 135.22	1,207.93 ± 114.67	1,179.61 ± 79.73	NS
Observed						
OTU	d10–11	617.56^b^ ± 92.31	754.14^a^ ± 45.35	659.58^b^ ± 50.62	708.55^a^^b^ ± 85.25	** <0.05**
	d31–32	722.10 ± 80.76	762.98 ± 98.10	733.11 ± 63.25	712.33 ± 52.63	NS
PD whole tree	d10–11	40.13^a^^b^ ± 5.28	47.00^a^ ± 2.82	41.34^b^ ± 4.00	43.62^a^^b^ ± 5.67	** <0.05**
	d31–32	44.99 ± 4.95	47.97 ± 5.97	46.56 ± 3.93	43.75 ± 3.03	NS
Shannon	d10–11	5.92 ± 0.44	6.40 ± 0.22	6.21 ± 0.22	6.39 ± 0.37	NS
	d31–32	6.49 ± 0.22	6.57 ± 0.39	6.42 ± 0.47	6.42 ± 0.27	NS

*OTU, operational taxonomic unit; PD phylogenetic diversity*.

*Mean values (n = 8 animals) ± SD*.

a, b *Mean values within a row with unlike superscript letters are significantly different (p < 0.05)*.

*The P-values < 0.05, p < 0.01, and p < 0.001 were considered as statistically significant, highly significant, and very highly significant*.

Sequencing of the 16S rRNA genes in colonic digesta produced a total of 3,900,165 quality-filtered sequences, with a mean number of 60,940 ± 29,645 reads (mean ± SD) per sample. Communities did not significantly differ between treatments, and no visual clustering could be detected on the weighted UniFrac PCoA plots, neither on d10–11 nor on d31–32 of the experiment (data not shown).

Colonic microbiota composition with a relative abundance of at least 1% was included in Table 7. At the first sampling time (d10–11), the main phyla observed were *Firmicutes, Bacteroidetes*, and *Proteobacteria*; only two genera significantly differed between treatments, which disappeared after the FDR correction. The relative abundance of *Faecalibacterium* spp. was significantly increased in the colon of piglets fed with CP2% in comparison to the other diets. The colonic abundance in *Anaerovibrio* spp. was greater with IN and CP0.2% pigs compared to control pigs. When extending the analysis to microbiota communities with a relative abundance over 0.1% of the total colonic microbiota (Supplementary Table 5), six genera significantly differed at this sampling time although the FDR correction did not reveal any difference. The population of *Ruminococcus* spp. was lower in the colon of CP0.2%- and IN-fed piglets in comparison to the CP2%-supplemented piglets. The inclusion of CP2% induced the highest abundances of *Lachnospira* spp. (followed by CP0.2%) and unclassified *Peptostreptococcaceae*. Unclassified *p-2534-18B5* (followed by CP0.2%) and unclassified *Enterobacteriaceae* levels were significantly increased under the IN treatment.

**Table 7:** Microbiota composition in the colon after feeding a diet supplemented IN (IN0.2%) and CP (CP0.2% and CP2%) from post-weaning day 5 onward, at sampling times on d10–11 and d31–32 post-weaning.

**Sampling time**	**Phylum / Genus**	***P*-value**	**FDR**	**Control**	**IN0.2%**	**CP0.2%**	**CP2%**	**SEM**
d10–11	***Bacteroidetes***	NS	NS	26.9	25.2	26.6	25.7	1.4
	Unclassified *Paraprevotellaceae*	NS	NS	0.7	1.3	1.5	1.9	0.3
	*Prevotella*	NS	NS	23.7	21	22.9	21.7	1.7
	*S24-7*	NS	NS	1.5	1.3	1	1.1	0.5
	***Firmicutes***	NS	NS	70.3	71.9	70.3	72	1.7
	*Lactobacillus*	NS	NS	26.5	23.3	25.1	21.1	3.7
	*Streptococcus*	NS	NS	1.2	0.9	0.8	0.9	0.5
	Unclassified *Clostridiales*	NS	NS	2	3.3	3	2.7	0.3
	Unclassified *Clostridiaceae*	NS	NS	1.2	4.4	1.8	3.5	1
	*SMB53*	NS	NS	0.3	1.3	0.3	0.6	0.3
	Unclassified *Lachnospiraceae*	NS	NS	3.6	3.9	3.9	3.7	0.3
	*Blautia*	NS	NS	4.4	3.5	3.1	4.6	0.6
	*Coprococcus*	NS	NS	0.8	1.3	1	1.2	0.2
	*Roseburia*	NS	NS	4.5	6.9	6.1	6.4	1
	*Lachnospiraceae* Other	NS	NS	0.8	1.2	1	1.3	0.2
	Unclassified *Ruminococcaceae*	NS	NS	6.9	7.2	6.5	8.9	0.8
	*Faecalibacterium*	**0.04**	NS	2.4 ^b^	2.7 ^b^	2.4 ^b^	3.6 ^a^	0.4
	*Ruminococcus* OTU2	NS	NS	1.5	1.8	1.5	1.5	0.3
	*Anaerovibrio*	**0.04**	NS	1.3 ^b^	2.2 ^a^	3.2 ^a^	1.5 ^ab^	0.5
	*Megasphaera*	NS	NS	3.9	1.3	1.9	2	0.6
	*Phascolarctobacterium*	NS	NS	1.1	0.9	1.1	1.4	0.2
	*Eubacterium*	NS	NS	1	0.6	0.7	1	0.2
	*Catenibacterium*	NS	NS	1.1	0.2	1	0.7	0.5
	***Proteobacteria***	NS	NS	2	1.4	2.2	1.1	0.5
	*Succinivibrio*	NS	NS	1.1	0.3	1.1	0.2	0.4
d31–32	***Bacteroidetes***	NS	NS	27.5	25.9	27.2	27.5	1.2
	Unclassified *Bacteroidales*	NS	NS	0.8	1.2	1.2	0.8	0.2
	Unclassified *Paraprevotellaceae*	NS	NS	1.7	1.5	1.2	1.5	0.3
	*Prevotella*	NS	NS	23.4	21.1	23.3	23.5	1.3
	*S24-7*	NS	NS	1.1	1.4	1.1	1.4	0.3
	***Cyanobacteria***	**0.00**	**0.01**	1.3 ^a^	0.5 ^b^	0.9 ^ab^	0.3 ^b^	0.2
	Unclassified *2YS2*	**0.00**	**0.04**	1.3 ^a^	0.5 ^b^	0.9 ^ab^	0.3 ^b^	0.2
	***Firmicutes***	NS	NS	68.7	71.8	70	70.7	1.5
	*Lactobacillus*	NS	NS	11.6	15.8	13.1	17.4	4.1
	*Streptococcus*	NS	NS	2	1.4	2.3	3.7	0.9
	Unclassified *Clostridiales*	NS	NS	3.6	2.8	3.8	2.8	0.4
	Unclassified *Clostridiaceae*	NS	NS	7.6	7.1	7.9	4.4	1.7
	*SMB53*	NS	NS	1.2	1	1.4	0.7	0.3
	Unclassified *Lachnospiraceae*	**0.02**	NS	4.9 ^a^	4.6 ^a^	3.8 ^ab^	3.4 ^b^	0.4
	*Blautia*	NS	NS	3.4	3.7	2.6	3.3	0.4
	*Coprococcus*	NS	NS	1.3	1.3	1.2	1.1	0.1
	*Dorea*	NS	NS	0.9	1.1	0.7	1	0.2
	*Roseburia*	NS	NS	6.9	5.3	7.5	7	1
	*Lachnospiraceae* Other	NS	NS	1.6	1.4	1.3	1.6	0.2
	Unclassified *Ruminococcaceae*	NS	NS	7.5	10.2	8.2	8.5	0.7
	*Faecalibacterium*	**0.03**	NS	1.8 ^b^	2.4 ^ab^	2.2 ^b^	3.2 ^a^	0.4
	*Ruminococcus* OTU2	NS	NS	1.9	1.6	1.7	1.6	0.2
	*Anaerovibrio*	NS	NS	2	1.5	1.8	1.4	0.4
	*Megasphaera*	**0.03**	NS	1.0 ^b^	1.4 ^b^	1.4 ^b^	2.2 ^a^	0.3
	*Phascolarctobacterium*	NS	NS	1.1	1.4	1.3	1.1	0.1
	*Eubacterium*	NS	NS	1.4	0.8	0.9	0.8	0.4
	*Bulleidia*	**0.01**	NS	0.5 ^b^	1.3 ^a^	0.9 ^a^	0.3 ^b^	0.3
	***Proteobacteria***	NS	NS	1	0.6	0.7	0.6	0.3

*OTU, operational taxonomic unit*.

*The microbiota composition is expressed as a percentage (%) of the total microbiota*.

*Only genera and phyla with a relative abundance ≥1% for one or more treatment(s) were included in this table*.

*Mean values (n = 8 animals)*.

a, b *Mean values within a row with unlike superscript letters are significantly different (p < 0.05)*.

On the second sampling time (d31–32), colonic bacterial sequences present over 1% of the total microbiota predominantly included the *Firmicutes, Bacteroidetes, Cyanobacteria*, and *Proteobacteria* phyla (Table 7). The abundance in colonic *Cyanobacteria* was significantly affected by treatments with control piglets, showing greater proportions of the phylum than CP2%- and IN-fed piglets. Five genera significantly differed in the colon of pigs administered with the treatments on d31–32, considering a relative abundance of over 1% of the total microbiota (Table 7). The FDR correction did not reveal any difference between diets except for the *Cyanobacteria* phylum and unclassified *2YS2* (*p* < 0.05). Unclassified *2YS2* belonging to the *Cyanobacteria* phylum was significantly lower with CP2% and IN treatments compared to the control feed. Decreased levels of unclassified *Lachnospiraceae* were found in the colon of piglets offered CP2% in comparison to the control and IN-fed piglets. CP2% induced a higher abundance of *Faecalibacterium* spp., significantly differing from control and CP0.2% treatments. The population of *Megasphaera* spp. was increased by CP2% compared to the other treatments. The *Bulleidia* spp. the genus was present in higher proportions in the colon of IN and CP0.2%-fed piglets compared to the other two treatments. Considering genera present with a relative abundance of over 0.1% of the total colonic microbiota, five genera significantly differed between treatments although the FDR correction did not reveal any difference on d31–32 (Supplementary Table 5). *Enterococcus* spp. and unclassified *Christensenellaceae* were more abundant in the colon of piglets under IN supplementation compared to the other treatments. The colonic population of *Lachnospira* spp. was significantly higher with CP2% compared with IN and control diets. The genera *Clostridium* spp. and *Shuttleworthia* spp. were present in the lowest proportions with the CP2% treatment.

### Experiment 2

#### *In vivo* Intestinal Permeability

At 7 days after dietary treatment, piglets showed lower permeability by decreased serum xylose concentrations when fed with IN (0.45 ± 0.03 mmol/L) in comparison to control (0.69 ± 0.11 mmol/L), CP0.2% (0.77 ± 0.10 mmol/L), and CP2% (0.83 ± 0.14 mmol/L) treatments (*p* = 0.0868).

## Discussion

In this study, we investigated the prebiotic potential of CP as a dietary strategy to alleviate the weaning stress of piglets in comparison to IN and its use as a well-recognized prebiotic. Consequently, the study design included a control group and a group supplemented with the minimum effective dose of IN, as a positive control for desired effects (34). For the experimental groups, supplementation with CP was done at the same dose as IN (0.2%) and at 2%, since the previously obtained results *in vitro* had shown that CP, a complex product, could not achieve the same effects at the same dose as IN, a pure compound (35, 42). So, it was decided to use a 10-fold higher dose of CP. Prebiotics aim to provide beneficial effects on animal health through the effects on the microbiota and derived metabolites in the digesta. Therefore, to investigate the effects of CP on gastrointestinal health, we determined an array of genes related to apoptosis, inflammation, and barrier integrity in the colon tissue, where an alteration in SCFA and BCFA was observed.

During the investigation period, IN supplementation did not induce an effect on the growth performance of the piglets, which contrasts with the results obtained from the studies on IN supplementation during the suckling period (43). Despite the absence of IN effects on performance, some differences were found in the health-related parameters measured. Morphological measurements of the intestinal epithelium showed that IN increased villus height to crypt depth ratios in the duodenum, jejunum, and ileum, together with numerically decreased crypt depths, which might be attributable to a reduced cell division in the crypts. This would be supported by the downregulated *CASP1* expression on d10–11, acting as a key enzyme in programmed cell death and cell turnover rate although no other upregulations or downregulations of apoptotic genes were found in this study. The reduced acidic goblet cell absolute counts in the small intestine of the IN treatments suggest a reduction in mucus production resulting in a thinner mucus layer and are in accordance with the lower gene expression level of *MUC2* in the colonic tissues of pigs fed with IN on d31–32. However, the possible effect on the mucus layer, which was also observed for the CP treatments, may suggest a higher susceptibility to bacterial translocation (44). Furthermore, both CP treatments reduced the acidic goblet cell counts in the small intestine indicating a lower mucus production, in line with Hedemann et al. (45) and Swiech et al. (46). Interestingly, no modification of the mucin 1 gene (*MUC1*) was observed in this study, indicating the difficulty of data interpretation as not all parameters always go in the same line. However, in the theoretical event of bacterial translocation, a subsequent inflammatory response would occur, which was not observed in our study, seen the lack of differential colonic gene expression of the inflammatory genes. Furthermore, intestinal permeability *in vivo*, used as a marker for intestinal integrity, showed a tendency to decrease, implying increased integrity of the intestinal barrier, due to the IN treatment after 1 week and controversly lower levels of occludin on d31–32 *in vivo*. This would be in line with previous research showing that *in vitro* batch fermentation of IN using feces of the piglet produced increased levels of SCFA and butyrate, together with an upregulation of tight and adherens junction genes in IPEC-J2 cells (47). In general, IN fermentation is considered to occur mainly within the distal end of the small intestine in pigs (48), which may indicate a reduced capacity of the ingredient to modify porcine hindgut fermentation (34), and therefore, corresponds with our lack of response in the colonic and cecal metabolites and minor microbiota modulation following IN supplementation. Therefore, as several parameters pointed to a positive effect of IN, our data are in line with the general positive known effects of IN on gut health in livestock, such as increased weight and VH:CD ratio, reduced inflammation, improved gut barrier function, and a decrease of pathogenic bacteria abundance (30, 32, 33, 43). Moreover, IN supplementation has been shown to modulate immune responses, improve the gut barrier function, and favor antidiabetogenic microbiota in mice (49). Still, future investigations should also consider these targets on permeability and inflammation at the protein level for a better understanding of the underlying mechanisms.

The absorptive capacity and function of the intestine were shown to be affected by both doses of CP, as was the case for IN, with increased villus height to crypt depth ratios in the duodenum, jejunum, and ileum; which was related to decreased crypt depths. Similarly, a reduced mitotic count was observed in crypts after dietary pectin supplementation (41), and it has also been related to higher luminal viscosity (16), which was observed in the ileum of the CP2% treatment on d31–32. Conflicting results about the impact of pectin-rich ingredients on the small intestinal morphology have been documented in the weaned pigs (22, 41), but they could be explained by a dose-effect. At the animal level, the effect of CP was translated into temporary softer feces in week 2 for the CP2% treatment, and a higher FCR in the 1st week, without remaining or global effects for the entire investigation period. This higher FCR could also be explained by a higher villi height to crypt depth ratio, implying an increased absorptive surface with reduced proliferative activity.

Intestinal permeability was shown to remain unaltered when comparing between treatments, even though CP at 0.2% decreased mRNA levels of intestinal barrier integrity-related genes such as *claudin-3* and *MUC2* on d31–32 post-weaning in colonic tissue. The investigation of the inflammatory responses in the duodenum and jejunum might provide novel information and highlight correlations between these parameters.

At d10–11 post-weaning, after 5–6 days of CP supplementation, the 11 inflammation signaling target genes related to the AKT, MAPK, and TLR pathways remained unaffected by the different diets. The other 14 inflammatory target genes, including cytokines, chemokines, and defensins, did not differ between the control and the supplemented diets in colonic tissues at the first sampling time. The gene expression of *DEF*β*4a*, encoding for the β-defensin 2, was downregulated in colonic tissues following the IN and CP2% inclusion on d31–32 of the treatment supplementation, supposing a modest impact of these two treatments on the intestinal pro-inflammatory status. Other immunity-related genes, including the inflammation signaling pathway target genes but also cytokines and chemokines, remained unaffected by the treatments at the second sampling time, which is in line with Weber et al. (26) when supplementing the weaned piglets for a week with 7.5% CP.

Concerning the fermentation of the CP, we determined the targeted fermentation metabolites, namely SCFA, intermediary compounds, and BCFA, resulting from the fermentation of the bacteria residing in the ileum, cecum, and colon. None of these fermentation metabolites were affected by the dietary treatments, for both sampling times in the ileum nor the cecum, suggesting that the complex carbohydrate fraction of CP may shift the fermentation site to more distal regions of the digestive tract (17). Indeed, in the colonic digesta on d10–11, a significant increase in acetate proportions resulting from CP2% inclusion was observed, which is in concordance with the results of Moset et al. (50) and Pascoal et al. (51). Other studies reported acetate as the major end product of pectin fermentation (52), which is in line with our results. Concomitant with the rise in acetate proportions, BCFA levels were decreased in the colon of piglets fed with CP2% on d10–11, which is in agreement with the lower valerate concentrations found by Moset et al. (50) and Almeida et al. (25) in the weaned piglets. This result may suggest that the inclusion of CP in the diet of newly weaned piglets as an ingredient could reduce proteolytic fermentation in the hindgut shortly after weaning. The inclusion of CP0.2% did not cause any significant change in SCFA proportions, thereby the modulation of metabolites by CP was assumed to be dose-dependent.

For the determination of metabolites using LC-MS, a clear separation can be seen mainly between the CP groups and the other two groups, i.e., the control group and the IN group. At both sampling times, hesperidin and neohesperidin were identified by LC-MS as significantly more present in both CP treatments, which is not surprising as these flavonoids are the main compounds in citrus fruits (53). In a study where neohesperidin was given to piglets around the time of weaning, an enhanced expression of Na+/glucose cotransporter SGLT1 was observed, increasing the intestinal capacity to absorb glucose and avoiding nutrient malabsorption (54), and an increase in the cecal population abundance of *Lactobacillus* spp. (55). However, in our study, the supplementation of CP did not significantly alter the colonic abundance of *Lactobacillus* spp. although we did observe changes in colonic gut microbiota, which is consistent with the hypothesis that CP is mainly fermented in the hindgut (24). Nevertheless, the statistical significances disappeared after the FDR correction. Indeed, pigs offered CP2% showed higher colonic levels of *Faecalibacterium* spp. at both sampling times, *Megasphaera* spp. only at d10–11 sampling time and *Anaeribio spp*. only at d31–32 sampling in comparison to control counterparts, which are in line with those obtained by Bang et al. (56) in a study using pectin in human donors. Indeed, taxa of the *Lachnospiraceae* family (57) and the *Faecalibacterium* spp. genus (58, 59) were credited with pectin-degrading capacities, resulting in galacturonic acid and acetate productions (56). We suggest that the rise in acetate-producing bacterial populations like *Lachnospira* spp. may have led to an enhancement of other non-pectic saccharolytic bacteria, such as *Megasphaera* spp. and *Ruminococcus* spp., *via* cross-feeding processes. It should be emphasized that the CP0.2% treatment showed the lowest abundances of *Faecalibacterium* spp., *Megasphaera* spp., and *Ruminococcus* spp., suggesting, once again, a dose-dependent effect of CP.

The metabolites determined by GC-MS showed differences when looking at the top compounds between the control group and the treatment groups, and some interesting compounds were identified although only significant by *P*-value and becoming insignificant after the FDR correction. Octadecenoic acid was found in higher amounts in all treatment groups at d10–11, which is a compound generated by lactic acid bacteria in the gut and has been described to provide protection against oxidative stress cytotoxicity (60). At d10–11 sampling time, the compound 3-IAA was detected in all samples from CP-treatment groups and only in two to three samples in control and IN groups, which is a microbiota-derived tryptophan catabolite produced in the colon by bacteria such as *Lactobacillus* (61, 62), and it has been shown to reduce susceptibility to lipopolysaccharide (LPS) (61), to promote anti-inflammatory responses (63), and to be involved in the maintenance of intestinal immune cells *via* AhR signaling (62). Finally, pipecolic acid was identified at d31–32 sampling time in all individuals at higher amounts in control and CP0.2% treatment groups. Pipecolic acid is a bacterial-derived lysine catabolite that has been related to increased feed efficiency in pigs (64), which, from our results, could indicate a decreased proteolytic fermentation due to IN and CP2% treatments. Despite the absence of statistically significant differences between groups, the identified compounds proved to be of high interest, and further research would be necessary to draw conclusions.

In conclusion, CP modulated the fermentation processes in the hindgut, as seen by an enhancement of several health-related bacterial populations for both periods in the colon of the CP2% treatment, together with higher acetate and lower BCFA proportions. The altered small intestinal morphology and acidic goblet cell count for both IN- and CP-supplemented pigs demonstrate effects on the host that merit further investigation. Nevertheless, a challenging trial with *E. coli* could provide new insights into the preventive or curative properties of CP against pathogen infection, under the conditions found at weaning in commercial pig productions.

## Data Availability Statement

Raw sequences have been uploaded in the European Nucleotide Archive database (project number PRJEB38284).

## Ethics Statement

These animal experiments were approved by Animal Ethical Committee of the University of Liège and the University of Ghent.

## Author Contributions

JU, NE, JB, MS, GB, JW, and GP: conceptualization, methodology, supervision, project administration, and validation. JU, EA, KK, MS, ST, and MD: research conduction. NE and GB: funding acquisition and resources. JU, EA, and MS: investigation and writing—original draft. All authors: review and editing.

## Funding

This work was supported by the Research Foundation for Industry and Agriculture, FRIA-FNRS, Belgium (Grant No. ID33848511), and the Fonds de la Recherche Scientifique-FNRS (Grant No. 31248729).

## Conflict of Interest

ST, MD, and GB were employed by company Royal Agrifirm Group. The remaining authors declare that the research was conducted in the absence of any commercial or financial relationships that could be construed as a potential conflict of interest. The handling editor declared a past co-authorship with one of the authors EA.

## Publisher's Note

All claims expressed in this article are solely those of the authors and do not necessarily represent those of their affiliated organizations, or those of the publisher, the editors and the reviewers. Any product that may be evaluated in this article, or claim that may be made by its manufacturer, is not guaranteed or endorsed by the publisher.
