# Supplementary material for: Impact of Citrus Pulp or Inulin on Intestinal Microbiota and Metabolites, Barrier, and Immune Function of Weaned Piglets
Source: Front Nutr. 2021 Dec 3;8:650211. doi: 10.3389/fnut.2021.650211 (PMC8679862; doi:10.3389/fnut.2021.650211)
Supplement: Supplementary file 1 [file Data_Sheet_1.pdf]

Supplementary Material

1 Supplementary tables

Supplementary table 1. Primer sequences for the gene expression of colonic tissue.

	Gene		Sequence	Accession no		Gene		Sequence	Accession no
Housekeeping genes	*ACTB*	F	CTACGTCGCCCTGGACTTC	XM_003124280.5	Inflammatory targets	*CXCL10*	F	CCCACATGTTGAGATCATTGC	NM_001008691.1
		R	GCAGCTCGTAGCTCTTCTCC				R	GCTTCTCTCTGTGTTCGAGGA	
	GAPDH	F	GATGGTGAAGGTCGGAGTGAA	XM_021091114.1		*DEFβ4a*	F	CAGGATTGAAGGGACCTGTT	AY506573.1
		R	GTGGAGGTCAATGAAGGGGT				R	CTTCACTTGGCCTGTGTGTC	
	HPRT1	F	AATTCTTTGCTGACCTGCTGGA	XM_021079503.1		*INFα*	F	CATTGCAGCTGAGTAGCACC	XM_021062981.1
		R	TCCACCAATTACTTTTATATCGCCC				R	CAGAAGCATCTGCAAGGTTCC	
	PPIA	F	GGGACCTGGAAACCAAGAAGTG	XM_013985800.2		*INFβ*	F	TTCGAGGTCCCTGAGGAGATT	NM_001003923.1
		R	ACTTTGTCTGCAAACAGCTCCAATC				R	GCTGGAGCATCTCGTGGATAA	
	RPL13α	F	ATTGTGGCCAAGCAGGTACT	XM_013998640.2		*IL1α*	F	GTTTCCACGCGGAGAGTGAG	XM_005655198.3
		R	AATTGCCAGAAATGTTGATGC				R	AAAGAGGTCAGGGACTTTGGC	
	RPL32	F	GCTTGAAGTGCTGCTAATGTG	XM_021068582.1		*IL1β*	F	CCAAAGAGGGACATGGAGAA	XM_021085847.1
		R	GGATTGGTGACCCTGATGGC				R	GGGCTTTTGTTCTGCTTGAG	
	RPL4	F	GAGAAACCGTCGCCGAATCC	XM_005659862.3		*IL6*	F	TGGGTTCAATCAGGAGACCT	NM_001252429.1
		R	CCCACCAGGAGCAAGTTTCAA				R	CAGCCTCGACATTTCCCTTA	
	TBP	F	CGGACCACCGCACTGATATT	XM_021085483.1		*IL8*	F	GACTTCCAAACTGGCTGTTGC	JF906514.1
		R	TTCTTCACTCTTGGCTCCCG				R	ATTTGGGGTGGAAAGGTGTG	
	YWHAZ	F	TTGTAGGAGCCCGTAGGTCA	NM_001315726.1		*IL18*	F	CTGAAAACGATGAAGACCTGGA	XM_005667326.2
		R	AGCACCTTCCGTCTTTTGCT				R	CCTCAAACACGGCTTGATGTC	
Inflammation signalling pathway targets	*AKT1*	F	CTAAGCCCAAACACCGCGT	XM_021081499.1		*INFγ*	F	TCACTGATGGCTTTGCGCT	NM_213948.1
		R	TCAGGATCTTCATGGCGTAGT				R	GGCCATTCAAAGGAGCATGGA	
	IKKβ	F	TTGTAGCAAGGTCCGTGGTC	NM_001099935.1		*ILRN1*	F	TGCCTGTCCTGTGTCAAGTC	NM_214262.1
		R	CAGCCACGAGGTCTTCACTT				R	GTCCTGCTCGCTGTTCTTTC	
	LBP	F	TTCTGCTCACGTCCATCCTG	NM_001128435.1		*MCP1*	F	CTCACTGCAGCCACCTTCT	NM_214214.1
		R	CCATTGAAGTCGGGGAGTGT				R	CACTTGCTGCTGGTGACTCT	
	MAPK14	F	TACCCGAGCGTTACCAGAAC	XM_001929490.6		*PPARγ*	F	ACAGCGACCTGGCGATATTTA	XM_005669784.3
		R	TTCACTGCAACACGTAACCCA				R	GAGGACTCTGGGTGGTTCAA	
	MyD88	F	GCATCACCATTCGAGATGACC	NM_001099923.1		*TNFα*	F	TCTGCCTACTGCACTTCGAG	NM_214022.1
		R	TCCTGCACAAACTGGGTATCG				R	GTTGATGCTCAAGGGGCCA	
	NF-κB1	F	AAGAAGTCCTACCCTCAGGTCA	NM_001048232.1	Barrier-integrity targets	*CDH1*	F	AGCCCTGCAATCCTGGCTTT	NM_001163060.1
		R	CAGTGACAGTGCAGATCCCA				R	AGAAACATAGACCGTCCTTGGC	
	NOD1	F	GTCGTCAACACCGATCCAGT	NM_001114277.1		*Claudin-1*	F	GGTGACAACATTGTGACGGC	NM_001244539.1
		R	CCTCCTTCTGGGCATAGCAC				R	TACCATCAAGGCACGGGTTG	
	TANK	F	GACCGGTCTGATGCACCTTT	XM_021075290.1		*Claudin-3*	F	TATCACAGCGCGGATCACC	NM_001160075.1
		R	TCTGAGCCTGTGCCACTTAG				R	CTCTGCACCACGCAGTTCAT	
	TLR2	F	GTTTTACGGAAATTGTGAAACTG	XM_005653576.3		*Claudin-4*	F	CTTCATCGGCAGCAACATCG	XM_013995522.2
		R	TCCACATTACCGAGGGATTT				R	CGAGTCGTACACCTTGCACT	
	TLR4	F	ATGATTCCTCGCATCCGCCT	NM_001113039.2		*JAMb*	F	GATGCCCAAAGCACCAACAG	XM_021070861.1
		R	AATTCAGCTCCATGCATTGGTAA				R	TGTCCGACAGAATTACGGGC	
	TLR5	F	GAGCATTGACAGGGAAGCCT	NM_001348771.1		*MARVELD2*	F	CTCAGCCCCGCCATTACCTG	NM_001128435.1
		R	TCTGGGTGCAAGAATGCGAT				R	TAGAGGTGATGTGCTGTTGCC	
Apoptosis-related	*BAX*	F	CCCAGAGGCGGGGTTTCAT	XM_013998624.2		*MUC1*	F	GGATTTCTGAATTGTTTTTGCAG	XM_021089728.1
		R	CAATGCGCTTGAGACACTCG				R	ACTGTCTTGGAAGGCCAGAA	
	CASP1	F	GTTATTCGGAAAGGGCCCCA	NM_214162.1		*MUC2*	F	GGACGACACCATCTACCTCAC	XM_021082584.1
		R	CACCGCCTGGGATTCTTGTA				R	AGGCCAGCTCGGGAATAGA	
	CASP3	F	AAGCAAATCAATGGACTCTGGAA	NM_214131.1		*Occludin*	F	AACGTATTTATGACGAGCAGCCC	NM_001163647.2
		R	TTGCAGCATCCACATCTGTACC				R	CACTTTCCCGTTGGACGAGTA	
	JUN	F	CTTTCCTCCTTCACGGTCCC	NM_213880.1		*ZO-1*	F	AAGGTCTGCCGAGACAACAG	XM_021098827.1
		R	CACTTCACGTGGGGTGAGTT				R	TCACAGTGTGGTAAGCGCAG	

Abbreviations: actin beta (***ACTB***); serine/threonine-protein kinase 1 (***AKT1***); BCL2 associated X protein (***BAX***); caspase (***CASP***); E-cadherin (***CDH1***); C-X-C motif chemokine 10 (***CXCL10***); defensin beta (***DEFβ***); glyceraldehyde-3-phosphate dehydrogenase (***GAPDH***); hypoxanthine phosphoribosyltransferase 1 (***HPRT1***); interferon (***IFN***); inhibitor of nuclear factor kappa-B kinase (***IKKβ***); interleukin (***IL***); interleukin-1 receptor antagonist (***ILRN1***); junctional adhesion molecule (***JAM***); AP-1 transcription factor subunit (***JUN***); lipopolysaccharide binding protein (***LBP***); mitogen-activated protein kinase 14 (***MAPK14***); tricellulin (***MARVELD2***); monocyte chemoattractant protein 1 (***MCP-1***); mucin (***MUC***); myeloid differentiation primary response 88 (***MyD88***); nuclear factor-kappa B (***NF-κB***); nucleotide-binding oligomerisation domain-containing protein 1 (***NOD1***); peroxisome proliferator-activated receptor gamma (***PPARγ***); peptidylprolyl isomerase A (***PPIA***); ribosomal protein L (***RPL***); TRAF family member-associated NF-kappa-B activator (***TANK***); TATA box binding protein (***TBP***); toll-like receptor (***TLR***); tumour necrosis factor alpha (***TNFα***); tyrosine 3-monooxygenase/tryptophan 5-monooxygenase activation protein zeta (***YWHAZ***); zonula occludens-1 (***ZO-1***).

Supplementary table 2. Effect of inulin (IN) and citrus pulp (CP0.2% and CP2%) on the weekly growth performance from weaning (d1) and during four weeks (d28).

	Time	Control	IN0.2%	CP0.2%	CP2%	P-value treatment
**BWG** (kg)	d1-7	1.26 ± 0.09	1.21 ± 0.09	1.18 ± 0.06	1.06 ± 0.06	0.1029
	d8-14	1.86 ± 0.10	1.94 ± 0.13	1.94 ± 0.08	1.84 ± 0.07	0.6767
	d15-21	2.44 ± 0.16	2.53 ± 0.14	2.37 ± 0.13	2.33 ± 0.10	0.6130
	d21-28	3.43 ± 0.21	3.54 ± 0.18	3.45 ± 0.15	3.25 ± 0.17	0.5442
	d1-d28	9.01 ± 0.48	9.26 ± 0.49	8.94 ± 0.38	8.41 ± 0.28	0.3795
**FI** (kg)	d1-7	1.81 ± 0.08	1.74 ± 0.08	1.85 ± 0.05	1.75 ± 0.07	0.5193
	d8-14	2.90 ± 0.06	2.87 ± 0.16	2.98 ± 0.08	2.81 ± 0.08	0.6664
	d15-21	4.01 ± 0.12	4.01 ± 0.20	3.97 ± 0.14	3.89 ± 0.14	0.8431
	d21-28	5.55 ± 0.18	5.57 ± 0.30	5.31 ± 0.15	5.32 ± 0.20	0.6166
	d1-d28	14.26 ± 0.38	14.19 ± 0.70	14.11 ± 0.34	13.77 ± 0.32	0.7736
**FCR**	d1-7	1.45 [b] ± 0.06	1.46 [ab] ± 0.08	1.58 [ab] ± 0.06	1.66 [a] ± 0.05	**0.0409**
	d8-14	1.59 ± 0.08	1.50 ± 0.05	1.55 ± 0.05	1.54 ± 0.04	0.6148
	d15-21	1.69 ± 0.11	1.60 ± 0.05	1.69 ± 0.05	1.67 ± 0.03	0.7337
	d21-28	1.64 ± 0.06	1.58 ± 0.05	1.55 ± 0.04	1.67 ± 0.11	0.4762
	d1-d28	1.60 ± 0.07	1.54 ± 0.03	1.59 ± 0.04	1.64 ± 0.04	0.3376

Abbreviations: body weight gain (**BWG**); feed conversion ratio (**FCR**); feed intake (**FI**).
Mean values (n = 8 pens) ± SEM.
[a,b] Mean values within a row with unlike superscript letters are significantly different ($P<0.05$).

Supplementary table 3. Effect of inulin (IN0.2%) and citrus pulp (CP0.2% and CP2%) on thickness of the *muscularis mucosae* and *tela submucosa* and the *tunica muscularis* thickness in the duodenum, jejunum and ileum of piglets on d10-11 and d31-32 post-weaning.

Tissue	Parameter	Time	Control	IN0.2%	CP0.2%	CP2%	P-value
**Duodenum**	***Muscularis mucosae + Tela submucosa (µm)***	d10-11	40 ± 2	48 ± 3	39 ± 2	42 ± 2	0.0658
		d31-32	43 ± 2	40 ± 2	45 ± 2	46 ± 2	0.0721
	Tunica muscularis (µm)	d10-11	246 ± 3	242 ± 12	232 ± 6	236 ± 9	0.6968
		d31-32	265 ± 15	261 ± 12	291 ± 19	286 ± 11	0.3960
**Jejunum**	***Muscularis mucosae*** + ***Tela submucosa*** (µm)	d10-11	47 ± 3	43 ± 2	44 ± 1	43 ± 2	0.5053
		d31-32	47 ± 3	47 ± 4	50 ± 3	50 ± 4	0.8606
	Tunica muscularis (µm)	d10-11	204 ± 11	195 ± 7	212 ± 13	215 ± 13	0.6179
		d31-32	231 ± 10	240 ± 8	245 ± 9	255 ± 9	0.2730
**Ileum**	***Muscularis mucosae*** + ***Tela submucosa*** (µm)	d10-11	46 ± 2	45 ± 3	47 ± 4	44 ± 2	0.8487
		d31-32	58 ± 2	51 ± 4	58 ± 3	55 ± 4	0.3599
	Tunica muscularis (µm)	d10-11	243 ± 15	234 ± 24	241 ± 22	241 ± 12	0.9852
		d31-32	351 ± 28	315 ± 12	308 ± 12	358 ± 34	0.3561

Supplementary table 4. Effect of treatment (treat) with inulin (IN) and citrus pulp (CP0.2 % and CP2 %) on metabolite concentrations in the ileal and cecal content of piglets on d10-11 and d31-32 postweaning.

Time	Section	Treat	Lactate (mg g-1 OM)	Pyruvate (mg g-1 OM)	Succinate (mg g-1 OM)	Formate (mg g-1 OM)	SCFAs (mg g-1 OM)	Acetate (%)	Propionate (%)	Butyrate (%)	BCFAs (%)
d10-11	Ileum	Control	4.57 ± 1.72	0.01 ± 0.01	0.09 ± 0.04	0.11 ± 0.02	0.57 ± 0.1	8.72 ± 5.1	77.95 ± 9.67	0 ± 0	13.33 ± 8.2
		IN0.2%	4.43 ± 1.2	0.02 ± 0	0.1 ± 0.04	0.12 ± 0.02	0.83 ± 0.09	17.29 ± 7.31	61.86 ± 6.71	0.71 ± 0.71	20.14 ± 7.1
		CP0.2%	3.19 ± 1.31	0.02 ± 0	0.1 ± 0.04	0.13 ± 0.02	0.64 ± 0.11	8.22 ± 4.05	60.98 ± 5.29	4.02 ± 2.2	26.79 ± 5.98
		CP2%	3.46 ± 1.35	0.01 ± 0	0.07 ± 0.03	0.09 ± 0.01	0.88 ± 0.35	20.51 ± 6.26	58.93 ± 5.15	7.35 ± 3.84	13.21 ± 5.62
		P-value	0.8708	0.7146	0.9247	0.4402	0.6708	0.3716	0.239	0.123	0.4529
	Caecum	Control	0.6 ± 0.27	0.01 ± 0.01	0 ± 0	0 ± 0	7.65 ± 0.64	47 ± 2.3	30.51 ± 2.28	18.53 ± 1.86	3.96 ± 1.1
		IN0.2%	0.36 ± 0.13	0.02 ± 0	0 ± 0	0 ± 0	8.76 ± 0.31	48.34 ± 1.11	31.98 ± 1.06	18.16 ± 1.04	1.53 ± 0.62
		CP0.2%	0.31 ± 0.14	0 ± 0	0 ± 0	0 ± 0	8.88 ± 0.31	47.47 ± 1.75	33.89 ± 1.72	16.8 ± 1.55	1.84 ± 0.69
		CP2%	0.54 ± 0.35	0.01 ± 0.01	0 ± 0	0 ± 0	7.91 ± 0.79	52.65 ± 2.59	29.39 ± 1.21	16.04 ± 2.81	1.92 ± 0.84
		P-value	0.8128	0.4528	0.6803	0.7674	0.3159	0.203	0.2606	0.7799	0.1743
d31-32	Ileum	Control	1.94 ± 0.55	0.02 ± 0	0 ± 0	0.15 ± 0.03	0.84 ± 0.15	32.91 ± 6.27	47.81 ± 8.23	1.9 ± 1.9	17.38 ± 6.08
		IN0.2%	3.4 ± 1.25	0.02 ± 0	0.04 ± 0.03	0.15 ± 0.03	0.99 ± 0.15	27.81 ± 6.29	48.88 ± 11.23	2.89 ± 1.58	20.41 ± 9.19
		CP0.2%	4.84 ± 1.76	0.01 ± 0	0.04 ± 0.03	0.13 ± 0.03	0.93 ± 0.16	29.76 ± 9.41	50.49 ± 8.66	4.69 ± 2.35	15.06 ± 6.67
		CP2%	3.76 ± 1.4	0.01 ± 0.01	0.06 ± 0.03	0.14 ± 0.03	1.01 ± 0.18	20.03 ± 4.85	44.14 ± 7.53	4.45 ± 2.28	31.39 ± 8.38
		P-value	0.4895	0.7697	0.3937	0.962	0.8694	0.6441	0.969	0.7543	0.4916
	Caecum	Control	0.59 ± 0.56	0.01 ± 0	0.02 ± 0.02	0 ± 0	8.22 ± 0.78	48.22 ± 1.67	31.45 ± 1.5	19.64 ± 2.04	0.68 ± 0.39
		IN0.2%	1.01 ± 0.59	0.01 ± 0.01	0.04 ± 0.03	0 ± 0	8.87 ± 0.61	48.45 ± 2.54	30.9 ± 1.74	19.22 ± 2.7	1.42 ± 0.83
		CP0.2%	1.61 ± 0.95	0.02 ± 0	0 ± 0	0 ± 0	8.52 ± 0.75	50.05 ± 1.74	28.04 ± 0.66	20.45 ± 1.34	1.47 ± 0.74
		CP2%	1.35 ± 0.86	0.03 ± 0.01	0 ± 0	0.01 ± 0.01	7.45 ± 0.68	51.97 ± 1.87	28.65 ± 1.03	17.31 ± 1.41	2.06 ± 0.92
		P-value	0.7971	0.1622	0.2774	0.6617	0.5378	0.5259	0.2013	0.7094	0.635

SCFAs = total amount of short-chain fatty acids (acetic + propionic + i-butyric + butyric + i-valeric + valeric acids; expressed as mg g-1 OM); acetic, propionic and butyric acid proportions (expressed as % of total SCFAs).
BCFAs = branched-chain fatty acid proportions (i-butyric + i-valeric + valeric acids scaled to SCFAs, expressed as %).
Mean values (n = 8 animals) ± SEM.

Supplementary table 5. The composition of the colonic microbiota of piglets (relative abundance ≥0.1%) on d10-11 and d31-32 postweaning with significant effects of inulin (IN) and citrus pulp (CP0.2% and CP2%)

Sampling Time	Phylum / Genus	P-value	FDR	Control	IN0.2%	CP0.2%	CP2%	SEM
d10-11	Unclassified *p-2534-18B5*	**0.02**	NS	0.01 [ab]	0.13 [a]	0.02 [a]	0.00 [b]	0.04
	Ruminococcus OTU1	**0.04**	NS	0.11 [ab]	0.08 [b]	0.07 [b]	0.23 [a]	0.04
	Lachnospira	**0.04**	NS	0.20 [b]	0.30 [ab]	0.37 [a]	0.60 [a]	0.08
	Unclassified *Peptostreptococcaceae*	**0.03**	NS	0.34 [ab]	0.28 [b]	0.44 [ab]	0.53 [a]	0.08
	Unclassified *Enterobacteriaceae*	**0.03**	NS	0.08 [ab]	0.30 [a]	0.01 [b]	0.06 [ab]	0.08
d31-32	*Enterococcus*	**0.01**	NS	0.00 [b]	0.31 [a]	0.00 [b]	0.00 [b]	0.05
	Unclassified *Christensenellaceae*	**0.04**	NS	0.02 [b]	0.10 [a]	0.03 [b]	0.04 [b]	0.02
	Clostridium	**0.03**	NS	0.40 [a]	0.49 [a]	0.30 [ab]	0.22 [b]	0.08
	Lachnospira	**0.01**	NS	0.44 [b]	0.26 [b]	0.48 [ab]	0.71 [a]	0.08
	Shuttleworthia	**0.02**	NS	0.10 [a]	0.04 [a]	0.50 [a]	0.00 [b]	0.09

Abbreviation: operational taxonomic unit (**OTU**).
The microbiota composition is expressed as percentage (%) of the total microbiota. Only genera statistically different between treatments were included in this table. Only genera and phyla with a relative abundance ≥0.1% for one or more treatment(s) were included in this table.
Mean values (n = 8 animals). FDR: false discovery rate correction
[a,b] Mean values within a row with unlike superscript letters are significantly different (P<0.05).

2 Supplementary figures

(A)

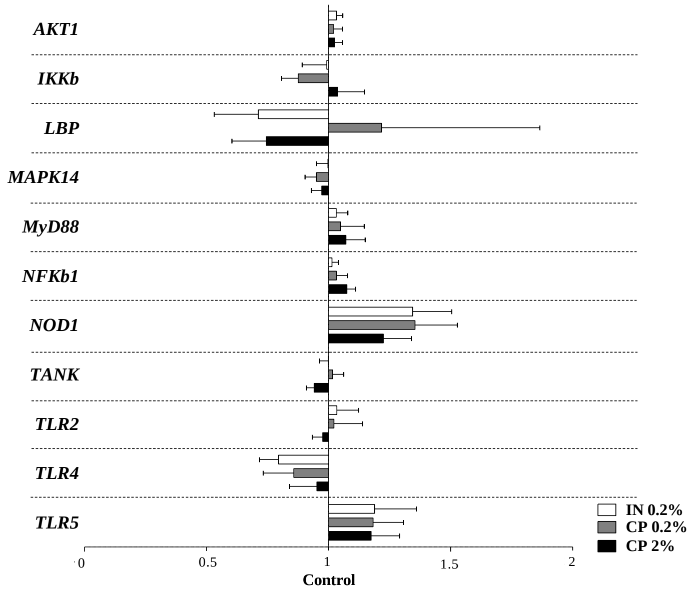

(B)

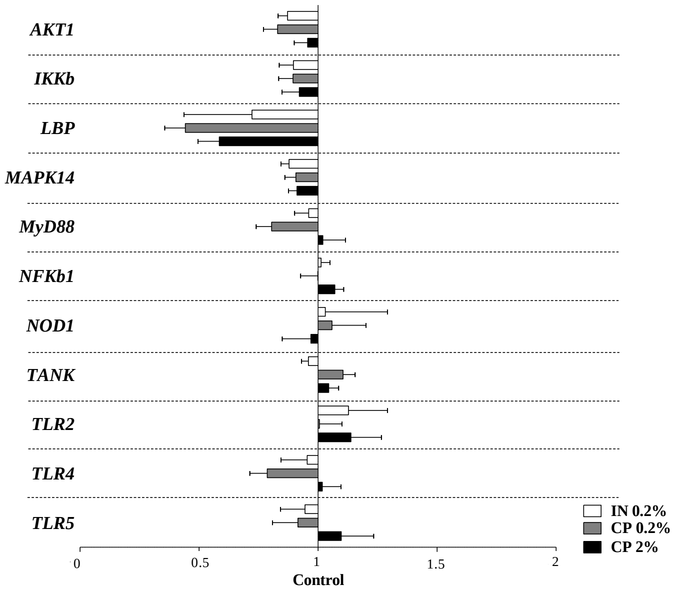

Supplementary figure 1. Effect of inulin (IN) and citrus pulp (CP0.2% and CP2%) on gene expressions of inflammation signaling pathway targets in colonic tissue on d10-11 (A) and on d31-32 (B) of the experiment.
Abbreviations: serine/threonine-protein kinase 1 (**AKT1**); inhibitor of nuclear factor kappa-B kinase (**IKKβ**); lipopolysaccharide binding protein (**LBP**); mitogen-activated protein kinase 14 (**MAPK14**); myeloid differentiation primary response 88 (**MyD88**); nuclear factor-kappa B (**NF-κB**); nucleotide-binding oligomerisation domain-containing protein 1 (**NOD1**); TRAF family member-associated NF-kappa-B activator (**TANK**); toll-like receptor (**TLR**).
Figures display the% of difference in comparison to the control treatment, considered as 1.
Mean values (n = 8 animals) ± SEM.

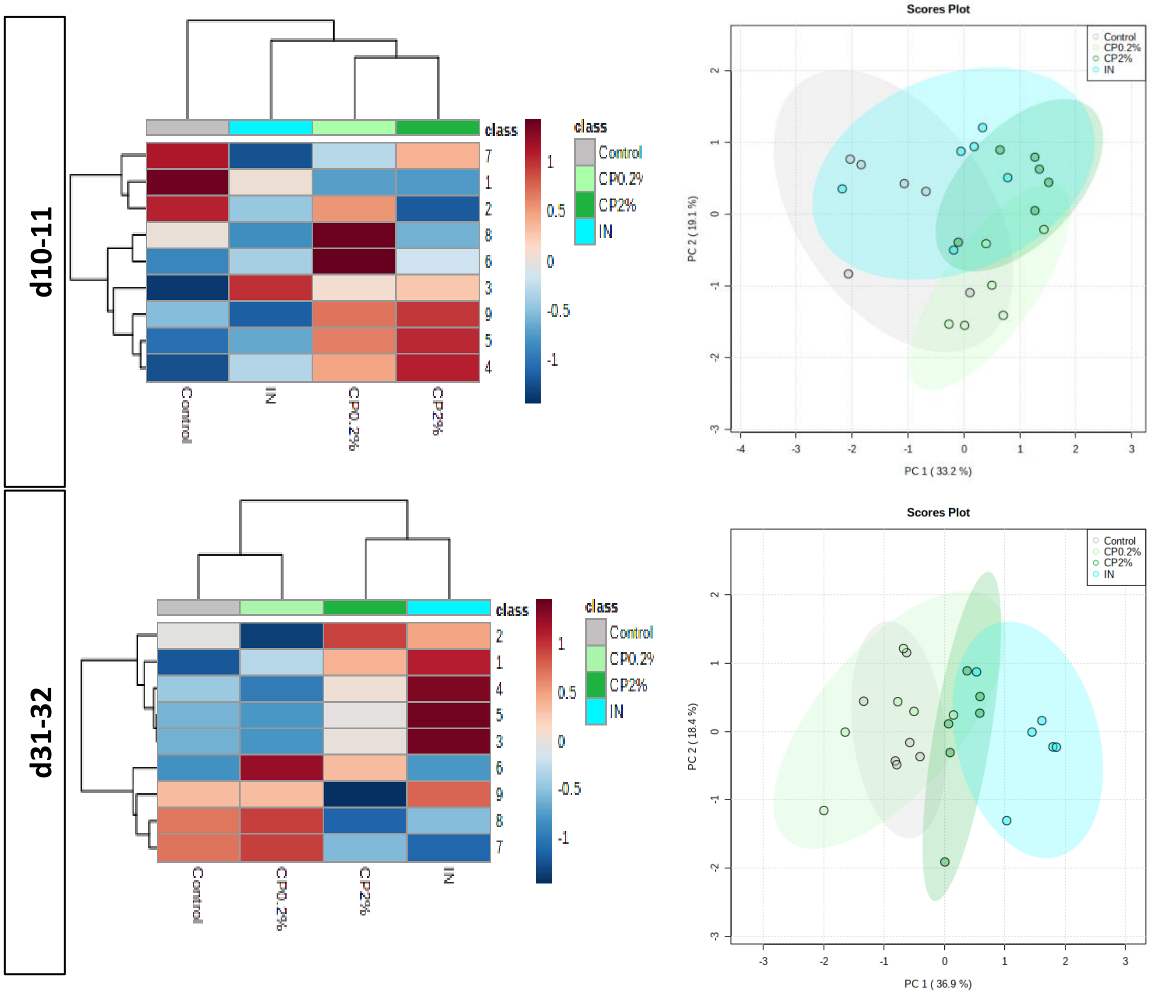

Supplementary figure 2. Heatmap (left) and PCA plots (right) showing the top most significant compound ions (p-value < 0.05) in the GC-MS analysis as shown in table 5, at sampling times d10-11 (top) and d31-32 (bottom). Numbers (1-9) refer to labels used in supplementary table 5 and present the different compounds. Groups compared were Control, inulin (IN) and citrus pulp (CP0.2% and CP2%). Each colored cell on the heatmap corresponds to the average concentration of the compound (row) per treatment group (column; n=6 per group).
